# Results and lessons from the Spironolactone To Prevent Cardiovascular Events in Early Stage Chronic Kidney Disease (STOP-CKD) randomised controlled trial

**DOI:** 10.1136/bmjopen-2015-010519

**Published:** 2016-02-25

**Authors:** Khai P Ng, Poorva Jain, Paramjit S Gill, Gurdip Heer, Jonathan N Townend, Nick Freemantle, Sheila Greenfield, Richard J McManus, Charles J Ferro

**Affiliations:** 1 Department of Renal Medicine, Queen Elizabeth Hospital Birmingham, Birmingham, UK; 2 Department of Primary Care Clinical Sciences, School of Health and Population Sciences, University of Birmingham, Birmingham, UK; 3 Department of Cardiology, Queen Elizabeth Hospital Birmingham, Birmingham, UK; 4 Department of Primary Care and Population Health, UCL Medical School, London, UK; 5 Nuffield Department of Primary Care Health Sciences, University of Oxford, Oxford, UK

**Keywords:** cardiovascular, chronic kidney disease, mineralocorticoid receptor antagonist, PRIMARY CARE, randomised trial, research recruitment

## Abstract

**Objectives:**

To determine whether low-dose spironolactone can safely lower arterial stiffness in patients with chronic kidney disease stage 3 in the primary care setting.

**Design:**

A multicentre, prospective, randomised, placebo-controlled, double-blinded study.

**Setting:**

11 primary care centres in South Birmingham, England.

**Participants:**

Adult patients with stage 3 chronic kidney disease. Main exclusion criteria were diagnosis of diabetes mellitus, chronic heart failure, atrial fibrillation, severe hypertension, systolic blood pressure <120 mm Hg or baseline serum potassium ≥5 mmol/L.

**Intervention:**

Eligible participants were randomised to receive either spironolactone 25 mg once daily, or matching placebo for an intended period of 40 weeks.

**Outcome measures:**

The primary end point was the change in arterial stiffness as measured by pulse wave velocity. Secondary outcome measures included the rate of hyperkalaemia, deterioration of renal function, barriers to participation and expected recruitment rates to a potential future hard end point study.

**Results:**

From the 11 practices serving a population of 112 462, there were 1598 (1.4%) patients identified as being eligible and were invited to participate. Of these, 134 (8.4%) attended the screening visit of which only 16 (1.0%) were eligible for randomisation. The main reasons for exclusion were low systolic blood pressure (<120 mm Hg: 40 patients) and high estimated glomerular filtration rate (≥60 mL/min/1.73 m^2^: 38 patients). The trial was considered unfeasible and was terminated early.

**Conclusions:**

We highlight some of the challenges in undertaking research in primary care including patient participation in trials. This study not only challenged our preconceptions, but also provided important learning for future research in this large and important group of patients.

**Trial registration number:**

ISRCTN80658312.

**Strengths and limitations of this study**

- This is the first reported randomised, placebo-controlled interventional trial of patients with stage 3 chronic kidney disease in the primary care setting in England.
- The strengths of the study are the original study design, setting and populations.
- The review of the list of potential participants by their corresponding general practitioners might have resulted in selection bias.
- The major limitation to this study was the poor recruitment which ultimately led to early termination of the study.
- Critical analysis and transparent reporting of research recruitment failure provide important learning for future research in this large and important group of patients.

## Introduction

Chronic kidney disease (CKD) is increasingly recognised as an independent risk factor for cardiovascular (CV) morbidity and mortality.^1–4^ Although the increased CV risk observed in dialysis patients is considerable, the global health burden of CV disease in the earlier stages of CKD is likely to be much greater given the high reported prevalence of up to 13% in developed countries.^5^ ^6^ In addition to an increased risk of vasculo-occlusive events such as myocardial infarction, patients with CKD also have an increased risk of cardiac arrhythmias and heart failure.^7^ ^8^ Increased arterial stiffness, leading to myocardial hypertrophy and fibrosis is thought to be a key mechanistic pathway in this pathophysiology of this increased CV risk.^7^ ^9–11^ Many of these abnormalities are indeed already evident in patients with only mild abnormalities of kidney function (estimated glomerular filtration rates (eGFRs): 30–90 mL/min/1.73 m^2^).^12–14^

Activation of the renin-angiotensin-aldosterone system (RAAS) is a key mediator of the arterial and cardiac changes observed in patients with CKD, as well as the increased CV risk associated with this condition.^15^ ^16^ Aldosterone is a mineralocorticoid, that is, a key effector of the RAAS. The mineralocorticoid receptor antagonist (MRA), spironolactone, used in low dose, has been shown to significantly lower arterial stiffness and left ventricular mass in patients with stage 3 CKD managed by nephrologists in secondary care.^17^ However, the majority (>90%) of patients with this level of kidney function are managed in primary care by their general practitioners (GP).^18^ ^19^ These patients tend to be older and have non-proteinuric renal diseases probably as a consequence of hypertension, renovascular disease and, possibly, normal ageing.^20^ ^21^ Furthermore, concerns about MRAs causing hyperkalaemia and worsening renal function might limit their future use in the primary care setting.

We, therefore, undertook a feasibility study to examine the actions of low-dose spironolactone on arterial stiffness in non-diabetic patients with stage 3 CKD managed in primary care. Secondary objectives included the rate of hyperkalaemia, deterioration of renal function, barriers to participation and expected recruitment rates to a potential future hard end point study.

## Subjects and methods

The study design has previously been described in detail (see online supplementary appendix 1).^22^ In brief, it was a multicentre, prospective, randomised, placebo-controlled, double-blind, parallel trial in non-diabetic patients with confirmed stage 3 CKD over 18 years of age in primary care. Patients with diabetes mellitus were excluded given that the pathophysiology of arterial stiffness is likely to be different, and they have a higher risk of hyperkalaemia.^23^ Diabetes would be expected to affect 20–30% of a community sample of CKD, and hence, would form a large subgroup within the trial. Thus, although diabetes is an important issue in CKD, we considered that this would be best explored in a separate study.

10.1136/bmjopen-2015-010519.supp1 Supplementary data

The GFR was estimated by the four-variable Modification of Diet in Renal Disease (MDRD) formula with serum creatinine recalibrated to be traceable to an isotope-derived mass spectroscopy method.^24^ The full inclusion and exclusion criteria are detailed in box 1. The UK National Institute for Health and Care Excellence (NICE) guidelines on CKD recommended systolic blood pressure (BP) (SBP) target range of 120–139 mm Hg, and diastolic BP (DBP) <90 mm Hg for patients with CKD.^25^ As spironolactone is known to have a BP-lowering effect, patients with SBP <120 mm Hg or postural hypotension were excluded from the study.^26^ In addition, due to the increased risk of hyperkalaemia associated with the use of spironolactone, patients with a serum potassium ≥5 mmol/L, or those already receiving both angiotensin converting enzyme inhibitor (ACEi) and angiotensin II receptor blocker (ARB) were also excluded from the study.^27^ Patients with uncontrolled severe hypertension (BP ≥180/110 mm Hg) were deemed inappropriate for the study as they required urgent antihypertensive treatment,^26^ and so were patients with urine albumin:creatinine ratio (uACR) ≥70 mg/mmol who should be referred and managed in a secondary care setting.

Box 1 **Inclusion and exclusion criteria of Spironolactone To Prevent Cardiovascular Events in Early Stage chronic kidney disease (STOP-CKD) study**

*Inclusion criteria*

- Age over 18 years
- Diagnosis of CKD stage 3 (modification of diet in renal disease (MDRD) estimated glomerular filtration rate (eGFR) 30–59 mL/min/1.73 m^2^, sustained for at least 90 days)

*Exclusion criteria*

- Diabetes mellitus
- Terminal disease or considered otherwise unsuitable by general practitioner
- Clinical diagnosis of chronic heart failure
- Atrial fibrillation
- Alcohol or drug abuse
- Inability to comply with trial medication and follow-up
- Documented previous hyperkalaemia or intolerance of spironolactone
- Documented Addisonian crisis or taking fludrocortisone
- Severe hypertension: blood pressure (BP) ≥180/110 mm Hg
- Systolic BP<120 mm Hg
- Recent acute kidney injury or hospital admission (within previous 6 weeks)
- Chronic diarrhoea
- Urine albumin:creatinine ratio (uACR)≥70 mg/mmol
- Serum potassium≥5 mmol/L on screening visit
- Concomitant co-trimoxazole medication
- Concomitant angiotensin converting enzyme inhibitor (ACEi) and angiotensin II receptor blocker (ARB) medication (dual-angiotensin blockade)
- Concomitant lithium medication
- Concomitant warfarin medication
- Pregnancy
- Breast feeding
- Planned major surgical intervention within 46 weeks of recruitment

Practices’ electronic patient records (EPR) were screened to identify all patients whose latest eGFRs were 30–59 mL/min/1.73 m^2^ in the preceding 12 months, and satisfied the study inclusion and exclusion criteria. Following review by individual patient's GPs to determine suitability for participation, potential participants were sent postal invitations addressed from their GPs (see online supplementary appendix 2). Non-responders were sent a second invitation 2 weeks later. Those expressing a willingness to participate were invited to attend a screening visit at their own general practice to confirm eligibility. Following this screening visit, eligible participants attended a randomisation visit within 2 weeks, and if still eligible were assigned 1:1 to receive either spironolactone 25 mg once daily orally, or an identical placebo for 40 weeks using a web-based randomisation system (see online supplementary appendix 1). Investigator, outcome assessors, data analysts and participants were all blinded to the treatment allocation. Patients were given a prescription to collect their study medication from their local community pharmacy. Arterial stiffness, as determined by carotid-femoral pulse wave velocity (cfPWV) was measured using the Vicorder system (Skidmore, Bristol, UK)^28^ at randomisation visit and 40 weeks. Blood pressure was measured using the BpTRU BPM-100 automated BP monitor, which was set to obtain six serial BP readings, at 1 min intervals.^29^ The mean office BP was derived from the 2nd and 3rd BP readings, whereas the mean BpTRU reading was derived from the 2nd to 6th readings.^30^ Postural hypotension was defined as a drop of SBP >20 mm Hg after a minute on standing.

10.1136/bmjopen-2015-010519.supp2 Supplementary data

The Primary Care Clinical Research and Trials Unit (PC-CRTU) at the University of Birmingham coordinated the study (trial registration number ISRCTN80658312).

### Sample size calculation

In a previous study of the effect of spironolactone, the SD of the change in cfPWV was 1.0 m/s in the active treatment group and 0.9 m/s in the control group.^31^ Hence, 100 participants in each arm would provide 90% power with an α value of 0.05 to detect a difference in change of cfPWV of 0.5 m/s between the active treatment and control groups. The study, therefore, intended to recruit 240 patients to account for an approximate dropout rate of 20%, which would result in at least 200 evaluable patients completing this trial.

### Statistical analysis

Statistical analyses were performed using SPSS V.20 (SPSS Inc, Chicago, Illinois, USA), and SAS V.9.4 (SAS Institute; Cary, North Carolina, USA). Numerical values are expressed as mean (SD) for parametric data, or median (IQR) for non-parametric data. Normality of the distribution of data was assessed by visual inspection of histogram and normal probability plot. Non-parametric data were log_e_-transformed before comparative analyses. Continuous data were compared using Student t tests.

Exploratory analyses were performed to identify any potential factors influencing patients’ willingness to participate. The information available on invited patients was limited to their age, gender, ethnicity, general practice and last recorded eGFR. These five factors were therefore assessed by binary logistic regression using a forced enter method with regard to their impact on the patient's research participation. Patients who expressed interest in participating were categorised as ‘willing invitees’, whereas patients who either did not respond to the invitation or replied but declined participation were grouped together as ‘non-willing invitees’. Patients’ gender (male/female) and ethnicity (white/others) were analysed as dichotomous data, whereas age and last recorded eGFR were analysed as continuous data. Supplementary analyses were performed with eGFR being dichotomised either into CKD stage 3a (eGFR: 45–59 mL/min/1.73 m^2^) and stage 3b (eGFR: 30–44 mL/min/1.73 m^2^) or into categories above or below the median of eGFR (54 mL/min/1.73 m^2^). Non-linearity of age and eGFR were examined using restricted cubic spline models. Models were selected on achieving a significant improvement in Akaike's Information Criterion. Statistical significance was defined as a two-tailed p value <0.05.

## Results

All 71 primary care practices within the former South Birmingham Primary Care Trust with more than 3000 patients registered were invited to participate. Eleven practices (15%) agreed to take part, with a total population of 112 462 (table 1). Electronic database searches identified 2044 potentially eligible patients. A further 446 (21.8%) patients were excluded by their GPs with the proportion excluded varying considerably (2.3–52.6%). Five of the 11 practices were known to be ‘research active’. There was no statistically significant difference in regard to proportions of patients excluded between ‘research-active’ practices compared to their counterparts (median 19 (IQR 10–47) vs 11 (IQR 4–28) %, p=0.2).

**Table 1:** Eleven recruiting practices’ population, prevalence of stage 3–5 CKD, numbers of patients invited, screened and randomised for STOP-CKD study

Practice	Practice population	Prevalence of stage 3–5 CKD* (%)	Patients eligible from computerised search (%)	Patients excluded by GP (%)†	Patients invited (%)	Patients replying (%)‡	Patients expressing interest (%)§	Patients attending screening visit (%)§	Patients randomised (%)§
#1¶	7501	4.72	260 (3.5)	49 (18.8)	211 (2.8)	105 (49.8)	37 (17.5)	22 (10.4)	3 (1.4)
#2¶	3838	1.86	38 (1.0)	20(52.6)	18 (0.5)	7 (38.9)	3 (16.7)	3 (16.7)	0
#3	27 025	4.82	360 (1.3)	183 (50.8)	177 (0.6)	102 (57.6)	21 (11.9)	15 (8.5)	1 (0.6)
#4	7113	3.58	179 (2.5)	7 (3.9)	172 (2.4)	81 (47.1)	20 (11.6)	12 (7.0)	2 (1.2)
#5	24 553	2.97	478 (1.9)	97 (20.3)	381 (1.6)	152 (39.9)	41 (10.8)	29 (7.6)	5 (1.3)
#6 ¶	8729	4.19	157 (1.8)	17 (10.8)	140 (1.6)	61 (43.6)	20 (14.3)	16 (11.4)	3 (2.1)
#7 ¶	5817	4.69	129 (2.2)	13 (10.1)	116 (2.0)	44 (37.9)	15 (12.9)	10 (8.6)	1 (0.9)
#8	4824	3.58	114 (2.4)	13 (11.4)	101 (2.1)	44 (43.6)	11 (10.9)	10 (9.9)	0
#9	9436	6.67	236 (2.5)	25 (10.6)	211 (2.2)	97 (46.0)	19 (9.0)	12 (5.7)	0
#10	7104	2.75	43 (0.6)	1 (2.3)	42 (0.6)	27 (64.3)	6 (14.3)	3 (7.1)	1 (2.4)
#11 ¶	6522	2.97	50 (0.8)	21 (42.0)	29 (0.4)	13 (44.8)	3 (10.3)	2 (6.9)	0
Total	112 462		2044	446	1598	733	196	134	16
Mean %		3.89	1.82	21.8	1.42				

% Indicates percentage of total practice population.

*Data obtained from Quality and Outcomes Framework 2013/2014 report.

^†^Indicates percentage of potentially eligible patients excluded by their general practitioner.

^§^Indicates percentage of patients invited.

^¶^Signify general practices which were research-active and had dedicated on-site practice research nurses.

‡Percentage of patients who replied to the STOP-CKD research invitations.

CKD, chronic kidney disease; GP, general practitioner; STOP-CKD, Spironolactone To Prevent Cardiovascular Events in Early Stage CKD.

### Invitation to study participation

**Figure 1:**
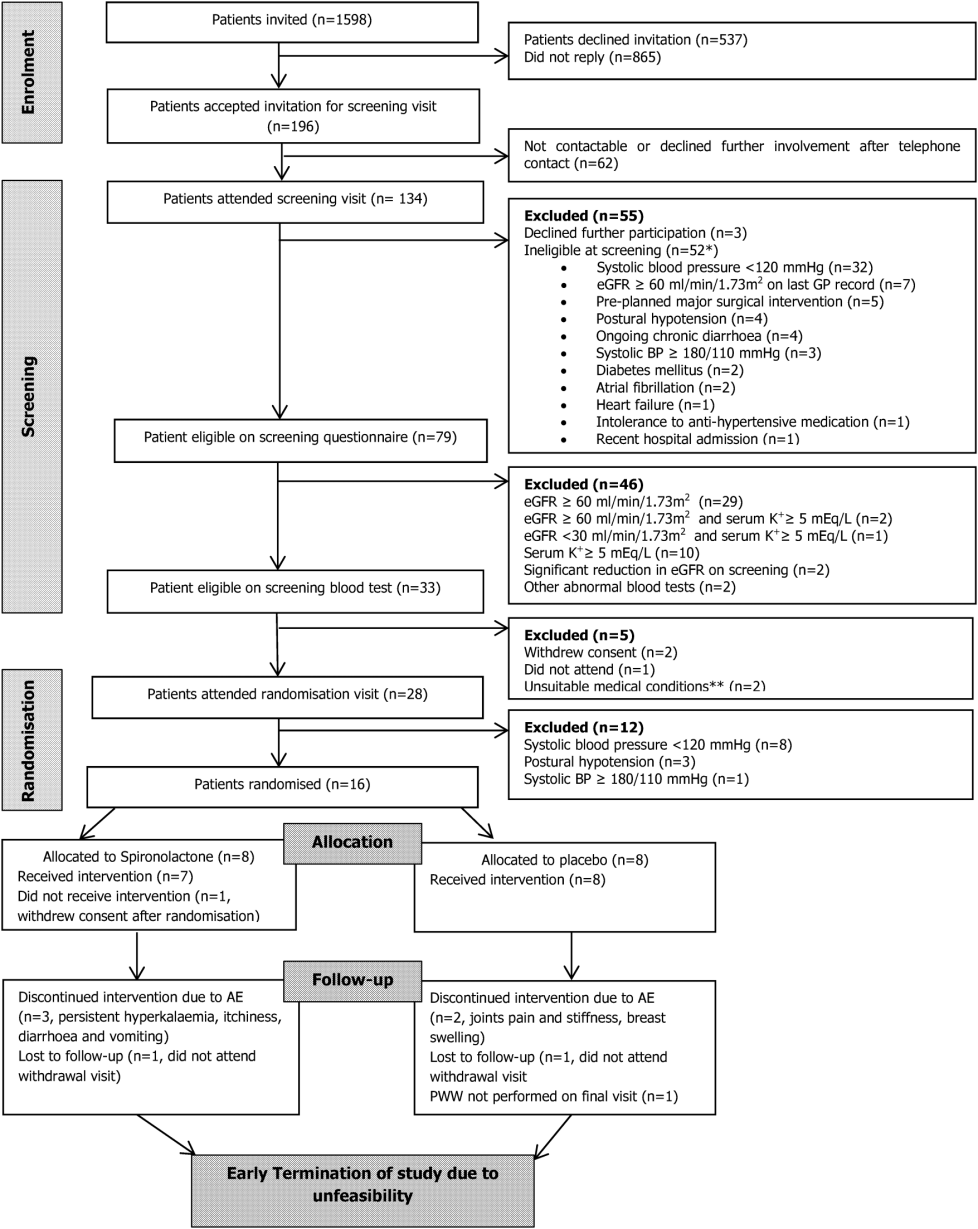
The CONSORT flow diagram of STOP-CKD study. *Some patients had multiple reasons for ineligibility. **Multiple adverse reaction to antihypertensive in the past and previous endovascular aortic aneurysm repair which would affect pulse wave velocity measurements. AE, adverse event; BP, blood pressure; eGFR, estimated glomerular filtration rate; STOP-CKD, Spironolactone To Prevent Cardiovascular Events in Early Stage Chronic Kidney Disease.

The trial started in July 2013. A total of 1598 invitation letters were sent out to all potentially eligible patients (figure 1). Sixty-three per cent were women with a mean age of 71 (SD 12) years, and a median eGFR of 53 (IQR 48–57) mL/min/1.73 m^2^. Most patients’ (84%) last eGFR readings were within the range of 45–59 mL/min/1.73 m^2^. The ethnicity was 83.4% white British, 3.4% black British, 3.3% South Asian, 1% mixed or other ethnicity, and 8.9% unknown.

### Patients’ response to study invitation letter

Responses were received from 733 patients (46%) who had a mean age of 73 (SD 11) years. Of these, 196 (12%) expressed interest in participating in the study. Percentages of those who were interested in participation ranged from 9% to 18% across the 11 practices (table 1).

Of the 537 patients who responded declining participation, 295 (55%) did not wish to take a new medication, 220 (41%) did not wish to be part of a research trial, 134 (25%) indicated that they did not have time to take part in the study, 86 (16%) did not wish to have further blood tests, 48 (9%) were unable to attend the surgery, 21 (4%) believed kidney problems were of no concern to them and 80 (15%) did not give a reason. Other reasons for non-participation detailed in the free-text area on the research reply slip included old age, poor mobility, presence of other health issues, concerns regarding the side effects of spironolactone, reluctance to take additional medication, work commitments, being carer for other family members, being away from home during trial period, as well as unawareness of CKD diagnosis.

**Figure 2:**
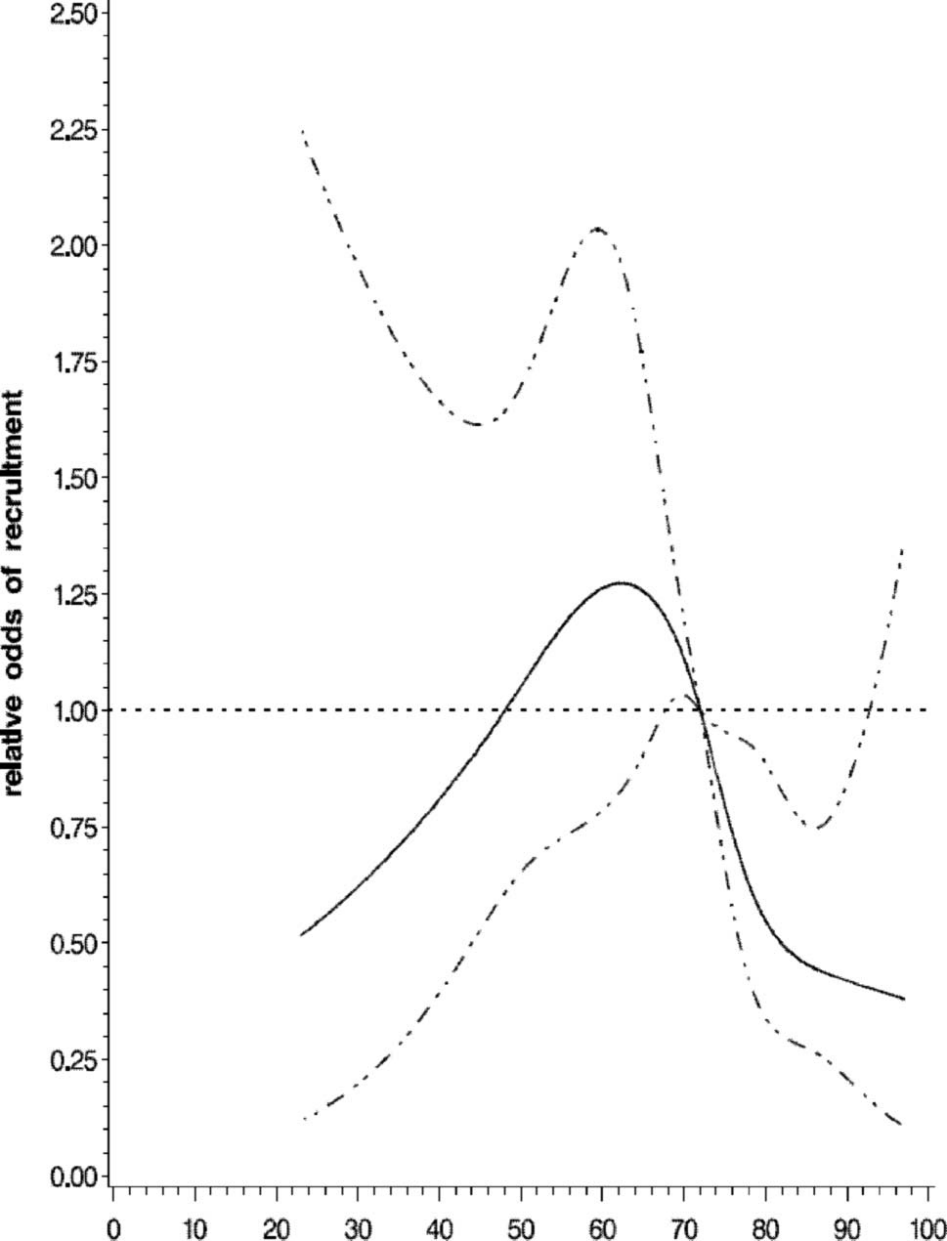
Relative odds and 95% CI of recruitment by age using restricted cubic spline. Solid line indicates estimate and dotted lines 95% CIs.

In a logistic regression model, age, male gender and coming from research-active practices were associated with a greater willingness to participate in the trial, whereas ethnicity and levels of eGFR were not predictive (table 2). Age was noticeably non-linear in relation to recruitment, with younger and older age associated with a lower likelihood of agreeing to participate in the study (figure 2).

**Table 2:** Logistic regression demonstrating factors associated with increased likelihood of patients’ willingness to participate in the trial (age as restricted cubic spline)

Variable	OR	Lower 95% CI	Upper 95% CI	p Value
Intercept	0.01931	0.00095	0.394	0.0103
eGFR	1.00513	0.98076	1.030	0.6827
**Male gender**	**1.36905**	**1.00544**	**1.864**	**0.0461**
White ethnicity	1.51474	0.96679	2.373	0.0699
**Research active practice**	**1.42223**	**1.04079**	**1.943**	**0.0270**
AGE	1.02677	0.97659	1.080	0.3014
AGE 1	0.93568	0.79398	1.103	0.4275
AGE 2	0.79572	0.15232	4.157	0.7865
AGE 3	3.30525	0.10044	108.771	0.5024
**p for non-linearity for age**				**0.0111**
**p for overall effect of age**				**0.0003**

Bold typeface highlights factors shown to have statistically significant association with increased likelihood of patients' willingness to participate in the trial (p<0.05).

eGFR, estimated glomerular filtration rate.

### Screening visit

Of the 196 patients who initially expressed an interest in participating in the study, 134 patients (69%) actually attended the screening visit. The characteristics of these patients are presented in table 3. The cause of CKD was unclear in the majority of the patients, and only 17 patients (13%) had a documented cause of CKD. The median last-recorded MDRD eGFR was 55 (IQR 51–57) mL/min/1.73 m^2^ with 88% within the range of 45–59 mL/min/1.73 m^2^.

**Table 3:** Baseline demographics, clinical characteristics, blood pressure measurements and biochemistry profiles of patients attended screening visit, and patients randomised to receive trial medication.

	Attended screening visit	Randomised into STOP-CKD study
**Number of patients**	**134**	**16**
Male gender, n. (%)	62 (46)	7 (44)
White ethnicity, n. (%)	125 (93)	16 (100)
Mean age (SD), years	68 (10)	71 (7)
**Medical history**, n (%)		
Hypertension	62 (46)	5 (31)
Hypercholesterolaemia	42 (31)	3 (19)
Coronary heart disease	17 (13)	1 (6)
Coronary artery bypass graft/angioplasty	13 (10)	0
Stroke/transient ischaemic attack	11 (8)	1 (6)
Peripheral vascular disease	8 (6)	0
Total number of comorbidities, median (IQR)	1 (0–2)	0 (0–1)
**Medications,** n (%)		
Antiplatelet agents	34 (25)	4 (25)
Lipid lowering agents	54 (40)	4 (25)
Use of antihypertensive agents	74 (55)	9 (56)
Diuretics	20 (15)	1 (6)
β-blockers	20 (15)	2 (13)
ACEi/ARB	48 (36)	5 (31)
Nitrates	5 (4)	0
Calcium channel blockers	21 (16)	4 (25)
α channel blockers	11 (8)	0
Patients not receiving any antihypertensive agents	60 (45)	7 (44)
**Smoking history,** n (%)		
Current smoker	8 (6)	1 (6)
Ex-smoker	55 (41)	7 (44)
Never smoker	71 (53)	8 (50)
**BP measurements**		
Office systolic BP, mean (SD), mm Hg	132 (19)	133 (10)
Office diastolic BP, mean (SD), mm Hg	79 (10)	78 (8)
Office systolic BP ≥140 or diastolic BP ≥90 mm Hg, n. (%)	47 (35)	10 (62)
Office BP within NICE CKD targets, n. (%)	54 (40)	6 (38)
Office systolic BP <120 mm Hg, n. (%)	34 (25)	0
**Number of patients**	**79**	**16**
Na^+^, mmol/L	141 (3)	142 (2)
K^+^, mmol/L	4.5 (0.6)	4.5 (0.4)
Urea, mg/dL	6.8 (2.0)	6.9 (1.4)
Creatinine, median (IQR), μmol/L	98 (85–112)	101 (86–121)
MDRD eGFR (median, IQR), mL/min/1.73 m^2^	57 (51–65)	54 (48–57)
CKD EPI eGFR (mean, SD), mL/min/1.73 m^2^	59 (12)	53 (7)
Urine ACR (median, IQR), mg/mmol	0.9 (0–2.0)	0.85 (0.08–1.95)
<3 mg/mmol, n. (%)	62 (79)	13 (81)
3–30 mg/mmol, n. (%)	16 (20)	3 (19)
>30 mg/mmol, n. (%)	1 (1)	0
Ca^+2^, mmol/L	2.38 (0.10)	2.38 (0.13)
Albumin, g/L	46 (2)	45 (1)
Total protein, g/L	72 (4)	71 (3)
Alkaline phosphatase, U/L	78 (25)	79 (17)
Alanine Aminotransferase, U/L	20 (8)	19 (7)

Bold typeface indicates categories of variables.

ACEi, ACE inhibitor; ACR, albumin:creatinine ratio; ARB, angiotensin II receptor blocker; BP, blood pressure; Ca^+2^, serum calcium; CKD, chronic kidney disease; EPI, Epidemiology Collaboration equation; eGFR, estimated glomerular filtration rate;; K^+^, serum potassium; MDRD, modification of diet in renal disease; Na^+^, serum sodium; NICE, National Institute for Health and Care Excellence; STOP-CKD, Spironolactone To Prevent Cardiovascular Events in Early Stage CKD.

In total, 52 (39%) patients were found to be ineligible for the study during the screening visit. The reasons for exclusion are listed in figure 1. The main cause for exclusion was low BP. Thirty-two patients had an office SBP lower than 120 mm Hg, with 16 patients receiving at least one antihypertensive agent, although five of these patients were known to have ischaemic heart disease, and thus, another potential indication for treatment with these agents other than hypertension. Of the 79 remaining eligible patients, a further 46 were excluded after the screening blood test (figure 1). The main reason for exclusion (31 patients) was having an eGFR >60 mL/min/1.73 m^2^.

### Randomisation visit

Of the 33 remaining eligible patients, 28 (85%) attended the randomisation visit (figure 1). A further 12 patients were excluded at this point. Eight had an office SBP <120 mm Hg, three had postural hypotension, and one had uncontrolled hypertension. Sixteen patients were randomised, and their baseline characteristics are shown in table 3.

### Early termination of study

In May 2014, the STOP-CKD study was terminated early because of futility. In order to achieve the original planned sample size of 240 patients, the projected number of primary care practices required to be involved in the study would be 145 practices covering a population of more than 1.5 million. After thorough discussion, the trial Data Monitoring and Ethics Committee, and the Trial Steering Committee collectively agreed that the study was not feasible with the allocated resources.

## Discussion

In the UK, as indeed in many countries, most patients with early-stage CKD are managed in primary care. Many observational studies have established that these patients have several differences compared with patients managed in secondary care.^32^ They tend to be older with a lower prevalence of proteinuria and more preserved eGFR.^32^ These differences are important if any intervention shown to be effective for the treatment of CKD in the minority of patients treated in secondary care is rolled out to the community. The STOP-CKD trial was an attempt to establish whether low-dose spironolactone, a treatment shown to be safe and effective in improving surrogate markers of CV risk in patients with CKD managed in secondary care, was equally safe and effective in patients with CKD managed in primary care. Although the study proved not to be feasible, there are several important findings and lessons that can be learnt from it to inform future interventional studies in this population.

### Estimating the number of patients needed

Assessing the number of patients needed to invite in order to recruit to the sample size is an essential but challenging requirement in planning any study. Recently, a Japanese study explored the use of information technology in predicting the success or failure of study recruitment.^33^ The study derived the eligible EPR index by dividing the number of eligible patients identified from the EPR by the target sample size. An EPR index of more than 1.7 was reported to have a sensitivity and specificity of approximately 70% and 100%, respectively, in predicting recruitment success. However, in spite of a much higher EPR index of 6.7 that should have predicted successful recruitment, the STOP-CKD study failed to reach its target sample size, suggesting that other recruitment issues were involved.

Following the EPR search, the number of patients eligible for invitation reduced considerably after GP review. The variation observed in the proportion of patients excluded by GPs across the practices suggests that there were large elements of subjectivity and inconsistency in this assessment. It is likely that many patients fulfilling the inclusion criteria were excluded at this stage. While the review of the list of potential participants by their corresponding GPs was well intentioned, significant selection-bias might have occurred during the process, and we suggest that in future studies, this step requires revision with clear and transparent criteria.

### Prevalence of CKD

In the UK, primary care physicians are required to keep a register of patients with stages 3–5 CKD.^34^ ^35^ Published data from the participating practices showed the average percentage of total patients on the CKD register was 3.89%, which is lower than the recently published reports from UK research databases of 5.15%^34^ and 5.9%,^36^ and marginally lower than that reported for all English practices over 2010–2012 of 4.3%.^37^ Nevertheless, it appears that the observed prevalence of CKD is much lower than the 10% figure which was the finding in previous epidemiological work in the UK^38^ and globally.^6^ ^39^ It has been suggested that the prevalence of CKD has been significantly overestimated by using a single serum creatinine measurement to define CKD,^40^ and this has been confirmed in a recent UK study using two creatinine measurements which reported a CKD prevalence of 3.9%.^34^

In order to increase patient inclusivity and bypass the issues of uncoded or miscoded CKD,^34^ we searched and shortlisted all patients with a latest recorded eGFR of 30–59 mL/min/1.73 m^2^ in the preceding 12 months. The eGFR test performed at the screening visit served as a confirmation of CKD diagnosis. Despite having an eGFR within 30–59 mL/min/1.73 m^2^ previously, 40% of such patients were excluded due to an eGFR >60 mL/min/1.73 m^2^ at screening, and therefore, by definition did not have CKD stage 3. Of those who fulfilled the biochemical eligibility criteria, most had only a modest reduction in eGFR, with a median eGFR of 54 mL/min/1.73 m^2^, and none were found to have significant levels of albuminuria.

### Blood pressure

The treatment of hypertension is still the cornerstone of management of CKD, both in terms of CKD progression and the reduction of CV risk.^41^ ^42^ In agreement with other studies, we found less than half the patients attending the screening visit achieved both the SBP and DBP target recommended by NICE CKD guidelines.^32^ ^38^ Among those with SBP ≥140 mm Hg or DBP ≥90 mm Hg, more than 40% were, in fact, not receiving any antihypertensive medication.

It has long been believed that lowering office/clinic BP to levels lower than 120/80 mm Hg is associated with worse outcomes and increased mortality, especially in the elderly.^43^ This is reflected in recent guidelines on the management of CKD that recommend BP not be lowered below these levels.^25^ ^44^ The results of the recent SPRINT trial challenge these guidelines,^45^ and future studies might consider the inclusion of such patients.

### Widening inclusion criteria

Faced with persistent recruitment difficulties, consideration to widen the STOP-CKD study eligibility criteria had been suggested. However, the eligibility criteria remained unchanged as each was believed to be essential not only in safeguarding patients’ safety, but also ensuring validity of the research study.

Patients with diabetes represent a significant subgroup of the CKD population. It is possible that as such patients have more regular contact with the healthcare professionals; they might potentially be more aware of their disease label and more willing to participate in the study. However, as patients with diabetes are known to have higher risk of hyperkalaemia and the pathophysiology of their increased arterial stiffness is likely to be different to those without, the inclusion of this subgroup of patients in this pilot study would result in a small study population too heterogeneous to effectively address the primary research question. Although the STOP-CKD study was unable to include patients with diabetes, the challenges faced by the study, and lessons learnt from it, have thus far been used to inform the ongoing Benefits of Aldosterone Receptor Antagonism in Chronic Kidney Disease (BARACK D) study,^46^ a large prospective, randomised, open blinded end point trial aiming to determine the effect of low-dose spironolactone on mortality and CV outcomes in patients with stage 3b CKD in primary care. Encompassing stage 3b CKD population with a minimal SBP of 100 mm Hg, and including those with type 2 DM; the participants’ criteria of BARACK D study, therefore, varies somewhat from that of the STOP-CKD. Its findings, assuming the trial successfully recruits, are therefore eagerly anticipated.

### Primary care practice recruitment strategies

Though we designed the STOP-CKD study to minimise any extra workload on the participating primary care practices, most practices declined the initial approach, and it took a lot of effort from the investigators to recruit the 11 practices that participated. In order to improve the quality and increase the quantity of primary care research in the UK, a ‘research-ready self-accreditation’ initiative to support general practices in meeting the legal requirements of the UK for carrying out research.^47^ Thus far, there are more than 1000 research-ready general practices in the UK.^48^ Our study demonstrated a significant positive influence of research-active practices on patients’ participation providing further support for these measures.

In addition, an integrated system which allows researchers to run complex searches over anonymised population-level health records, such as FARSITE, has proven to be a rapid method in testing research feasibility, providing accurate selection of a large patient population from a greater number of GP practices, facilitating administrative processes and, importantly, minimising research work-load for the practices.^49^ Innovative set-up of a more cohesive health informatics system looks to be the key in supporting and delivering faster and more effective research evidence of the real world for the future.^50^

### Patient recruitment strategies

Although the need for a robust evidence base for any intervention before it becomes accepted practice is now well established, there is surprisingly little evidence on how best to conduct an RCT.^51^ ^52^ Regulatory and ethical issues compelled us to contact potentially eligible patients by mailshot through their GPs. This is a notoriously inefficient and costly process with a large number of invitations needing to be sent to recruit the target number of patients. Two key reviews previously explored the value of various strategies in improving participants’ recruitment in research studies.^51^ ^53^ The STEPS study suggested that being flexible and robust in adapting to unexpected issues was important to ensure trials success,^53^ while in the systematic review by Treweek *et al*,^51^ telephone reminders to non-responders, opt-out rather than opt-in system of being contacted about the study, financial incentives and open designs all appeared to be effective strategies.

We suggest that an initial approach using telephone, text or email may yield better results, and that further research examining the acceptability and efficacy of initial recruitment strategies is of major importance. In addition, the effects of shortening patients’ information sheet,^54^ using computer pop-ups on patients’ electronic health records to highlight potential participants, minimising frequency of research visits and optimising the use of healthcare informatics for research data collection also worth exploring. Importantly, it is plausible that the issue of low awareness of CKD diagnosis among the patients^55^ might have negatively impacted on the recruitment of the STOP-CKD study. Efforts in developing wider recruitment strategies which focus on increasing potential participants’ awareness and understanding of CKD should therefore be considered.^56^ Suggested methods to improve CKD research recruitment in primary care are listed in box 2.

Box 2 **Suggested methods to improve chronic kidney disease (CKD) research recruitment in primary care**

- Open design
- Concise patient information sheet
- Minimise frequency of research visit
- Optimise use of routinely collected data via healthcare informatics
- Telephone, text or email as initial approach and reminder for non-responder
- Financial incentives/eliminating financial disincentives
- Computer pop-ups on healthcare records
- Increase disclosure of CKD diagnosis from healthcare providers to patients
- Improve patients’ understanding of CKD and its implications.

Our logistic regression model showed that younger and older patients were significantly less likely to participate. Thus, although we designed the study with broad inclusivity criteria, we still did not manage to recruit the ‘real-life CKD population’ which may reflect patients’ self-selection bias. Strategies to recruit these patients therefore need developing and testing in future studies.

## Conclusions

The STOP-CKD study was a non-age-restricted, investigator-led, feasibility RCT designed to inform a future larger, hard end point study in patients with CKD in primary care. The study highlighted the unique characteristics of the CKD population in primary care, which challenged our preconceived knowledge about the appropriate intervention and management of this sizeable group of patients. With the majority of interventional studies on patients with CKD thus far based in secondary care, there remains an urgent need to optimise the generalisability of future CKD research, especially in primary care. The experience and lessons learnt from this study provide important information for all CKD researchers to meticulously reflect on their future research aims, study design, choices of intervention, and most importantly, recruitment strategies. As Henry Ford once said, ‘failure is only the opportunity to begin again, only this time more wisely’.
