# Supplementary material for: Results and lessons from the Spironolactone To Prevent Cardiovascular Events in Early Stage Chronic Kidney Disease (STOP-CKD) randomised controlled trial
Source: BMJ Open. 2016 Feb 25;6(2):e010519. doi: 10.1136/bmjopen-2015-010519 (PMC4769397; doi:10.1136/bmjopen-2015-010519)
Supplement: Supplementary data [file bmjopen-2015-010519supp1.pdf]

UNIVERSITY OF BIRMINGHAM

PROTOCOL FOR CTIMP

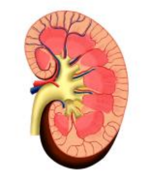

STOP-CKD

Spironolactone to Prevent Cardiovascular Events in Early Stage Chronic Kidney Disease: A Pilot Trial

Version 3.0

26th July 2012

Sponsor:	*University of Birmingham*
Chief Investigator:	*Dr Charles Ferro*
Study Co-ordination Centre:	*Primary Care Clinical Research and Trials Unit (PC-CRTU)*
EudraCT Number:	*2012-000654-55*
ISRCTN Number:	
PC-CRTU Number:	*CVD-001*

Sponsor

University of Birmingham
Edgbaston
Birmingham
B15 2TT

Chief Investigator

Dr Charles Ferro
Consultant Nephrologist and Senior Lecturer
Renal Unit
Queen Elizabeth Hospital Birmingham
Mindelsohn Way
Birmingham
B15 2WB

Tel: +44 (0) 121 3715839
Fax: +44 (0) 121 3715858
E-mail: charles.ferro@uhb.nhs.uk

Co-Investigators

Prof Richard McManus
Professor of Primary Care Cardiovascular Research
Department of Primary Care Health Sciences
University of Oxford
2nd Floor
23-38 Hythe Bridge Street
Oxford
OX1 2ET

Tel: + 44 (0)1865 617834
Fax: + 44 (0)1865 289287
Email: richard.mcmanus@phc.ox.ac.uk

Dr Paramjit Gill
Reader in Primary Care Research
Primary Care Clinical Sciences
School of Health and Population Sciences
College of Medical and Dental Sciences
University of Birmingham
Edgbaston, Birmingham
B15 2TT

Tel: + 44 (0)121 414 3758
Email: p.s.gill@bham.ac.uk

Dr John Townend
Consultant Cardiologist and Senior Lecturer
Cardiology Department
Queen Elizabeth Hospital
Birmingham
Mindelsohn Way
Birmingham
B15 2WB
Tel: + 44 (0) 121 6978476
Email: john.townend@uhb.nhs.uk

Dr Sheila Greenfield
Senior Lecturer in Medical Sociology
Primary Care Clinical Science
University of Birmingham
Edgbaston
Birmingham
B15 2TT
Tel: + 44 (0) 121 414 6493
Email: s.m.greenfield@bham.ac.uk

Dr Poorva Jain
Renal Medicine Research Fellow/Registrar in Nephrology
Primary Care Clinical Science
University of Birmingham
Edgbaston
Birmingham
B15 2TT
Tel: + 44 (0) 121 414 5643
Email: p.jain@bham.ac.uk

Research Fellow

For general queries, supply of trial documentation and collection of data, please contact:

Dr Khai Ping Ng
Renal Medicine Research Fellow
Queen Elizabeth Hospital Birmingham
Edgbaston
Mindelsohn Way
Birmingham
B15 2WB
Tel: + 44 (0) 800 9230329
Fax: + 44 (0) 121 414 8616
E-mail: k.p.ng@bham.ac.uk

Research Nurse

Gurdip Heer
Primary Care Research Nurse
Primary Care Clinical Science
University of Birmingham
Edgbaston
Birmingham
B15 2TT
Tel: +44 (0) 121 4146046
Emails: g.k.heer@bham.ac.uk

Study Administrator/Coordinator

Details to be confirmed

Statistician

Prof Nick Freemantle
Professor of Clinical Epidemiology & Biostatistics
Primary Care Clinical Sciences
University College London
Gower Street
London
WC1E 6BT

Tel: +44 (0)20 7794 0500 ex34756

Fax: +44 (0) 20 7794 1224

Email:
nicholas.freemantle@ucl.ac.uk

Coordinating Centre

Primary Care Clinical Research and Trials Unit (PC-CRTU)
University of Birmingham, UK

Funding

National Institute for Health Research
Grant ID.: PB-PG-0110-21226

This protocol describes the STOP CKD study and provides information about procedures for entering participants: it should not be used as a guide for the treatment of other participants. Every care has been taken in the drafting of this protocol, but corrections or amendments may be necessary. These will be circulated to investigators in the study, but centres entering participants for the first time are advised to contact the trials centre to confirm they have the most recent version. Problems relating to this trial should be referred, in the first instance, to the Study Co-ordinator.

This study will adhere to Good Clinical Practice and will be conducted in compliance with this protocol, the Data Protection Act, and other regulatory requirements, as appropriate.

Table of Contents

1 TRIAL SUMMARY 8
- 1.1 Title 8
- 1.2 Aims 8
- 1.3 Outcome Measures 8
- 1.4 Population 9
- 1.5 Number Of Sites 9
- 1.6 Eligibility 9
- 1.7 Treatment 9
- 1.8 Duration 9
- 1.9 Reference Diagram 10

2 INTRODUCTION 11
- 2.1 Background 11
- 2.2 Rationale For Current Trial 13

3 TRIAL OBJECTIVES 14

4 TRIAL DESIGN 14
- 4.1 Plan Of Investigation 14
- 4.2 Trial Outcome Measures 17
- 4.3 Study Timetable 18

5 PARTICIPANT ENTRY 19
- 5.1 Pre-Randomisation Evaluations 19
- 5.2 Sample Size 19
- 5.3 Inclusion Criteria 19
- 5.4 Exclusion Criteria 19
- 5.5 Withdrawal Criteria 20

6 STUDY PROCEDURES 22

7 RANDOMISATION AND ENROLMENT PROCEDURE 25
- 7.1 Randomisation Or Registration Practicalities 25
- 7.2 Unblinding 25

8 TREATMENTS 26
- 8.1 Treatment Arms 26

8.2 Dose Modifications For Toxicity 26
8.3 Pre-Medication 28
8.4 Interaction With Other Drugs 28
8.5 Dispensing And Accountability 30

9 TRIAL MANAGEMENT 31
9.1 Roles And Responsibilities 31
9.2 Trial Steering Committee 31
9.3 Data Monitoring Committee 31

10 PHARMACOVIGILANCE 32
10.1 Definitions 32
10.2 Causality 33
10.3 Reporting Procedures 33

11 ASSESSMENT AND FOLLOW-UP 35
11.1 Loss To Follow-Up 35
11.2 Trial Closure 35

12 STATISTICS AND DATA ANALYSIS 36
12.1 Statistical Plan 36
12.2 Analysis Plan 36
12.3 Data Handling, Record Keeping And Retention 36
12.4 Data Access and Quality Assurance 36
12.5 Case Report Forms 41

13 MONITORING 42
13.1 Risk Assessment 42
13.2 Monitoring At Study Co-ordination Centre 42
13.3 Monitoring At Local Site 43

14 REGULATORY ISSUES 44
14.1 Clinical Trial Authorisation (CTA) 44
14.2 Ethics Approval 44
14.3 Patient Consent 44
14.4 Confidentiality 45
14.5 Indemnity 45
14.6 Sponsor 45
14.7 Funding 45

14.8 Audits....45

15 FINANCIAL ARRANGEMENTS.... 46

15.1 Participant Payments....46

15.2 GP Payments....46

16 PUBLICATION.... 47

17 REFERENCES.... 48

18 PROTOCOL AMENDMENTS.... 52

Appendix A: STOP- CKD Hyperkalaemia Management Protocol

Appendix B: Patient Information Sheet

Appendix C: Consent Form

Appendix D: Low Potassium Diet Information Sheet

Appendix E: EQ5D-5L Form

Appendix F: Side Effect Questionnaire

Appendix G: Spironolactone medicine information

Appendix H: Role and responsibility

Appendix I: Trial Personnel Contact Sheet

Appendix J: Pharmacovigilance Flowchart

Appendix K: Serious Adverse Event Reporting Form

1 TRIAL SUMMARY

1.1 Acronym

STOP- CKD

Title

Spironolactone to Prevent Cardiovascular Events in Early Stage Chronic Kidney Disease (CKD): A Pilot Trial

1.2 Aims

Spironolactone, a mineralocorticoid receptor blocker (MRB) has been shown in double-blind, prospective, placebo-controlled trial in the secondary care setting to be associated with improved arterial stiffness and left ventricular mass and function. This suggests that spironolactone may reduce cardiovascular events in people with CKD. However, prior to a larger trial, further feasibility work is required to test procedures and outcomes in a primary care setting. This pilot study aims to examine:

- Patient and professionals' attitudes to spironolactone in CKD in a community setting and potential barriers exist to its use.
- The recruitment rate to the trial.
- The effect of spironolactone on arterial stiffness measured, by pulse wave velocity and pulse wave analysis, in primary care.
- The rate of spironolactone related hyperkalaemia and renal dysfunction in primary care and if it is affected by the method of analysis.

1.3 Outcome Measures

Primary end-point:

- Change in carotid-femoral pulse wave velocity between baseline and 40 weeks

Secondary end-points:

- Incidence of hyperkalaemia
- Change in blood pressure
- Change in estimated Glomerular Filtration Rate (eGFR)
- Incidence of hypotension (<100mmHg or >20mmHg systolic drop on standing)
- Incidence of side-effects
- Incidence of other adverse events or adverse reaction defined by the Medicines and Healthcare products Regulatory Agency (MHRA)
- Health status as measured by EQ5D
- Change in urinary albumin to creatinine ratio (ACR)
- Change of pulse wave analysis

1.4 Population

Patients registered in participating primary care practices within South Birmingham will be screened with a view to recruit 240 eligible patients with CKD stage 3 to this randomised controlled pilot study.

1.5 Number of Sites

Up to ten primary care practices within the South Birmingham will be involved in STOP-CKD trial.

1.6 Eligibility

Inclusion Criteria:

- Age over 18 years
- Diagnosis of CKD stage 3

Exclusion Criteria:

- Diabetes Mellitus
- Terminal disease or felt otherwise unsuitable by their general practitioner (GP)
- Chronic heart failure i.e. a clinical diagnosis or known ejection fraction (EF) <55%
- Atrial fibrillation
- Alcohol or drug abuse
- Inability to comply with trial medication and follow-up
- Documented previous hyperkalaemia or intolerance of spironolactone
- Documented Addisonian crisis and/or on fludrocortisone
- Severe hypertension: blood pressure ≥ 180/110 mmHg
- Systolic blood pressure < 120mmHg
- Recent acute kidney injury or hospital admission (within past 6 weeks)
- Chronic diarrhoea
- ACR ≥ 70mg/mmol (will require specialist referral if not already made)
- Serum potassium ≥ 5 mmol/L on screening blood test
- Concomittant co-trimoxazole medication
- Concomittant angiotensin-converting enzyme inhibitor AND angiotensin II receptor blocker medication
- Pregnancy

1.7 Treatment

240 patients will be recruited into the study and randomised between placebo and spironolactone 25mg daily.

1.8 Duration

All recruited patients will be receiving either placebo or spironolactone for 40 weeks. Patients will be followed-up during the duration of treatment and 6 weeks after discontinuation of trial medication (wash-out period). The total trial duration is 46 weeks.

1.9 Reference Diagram

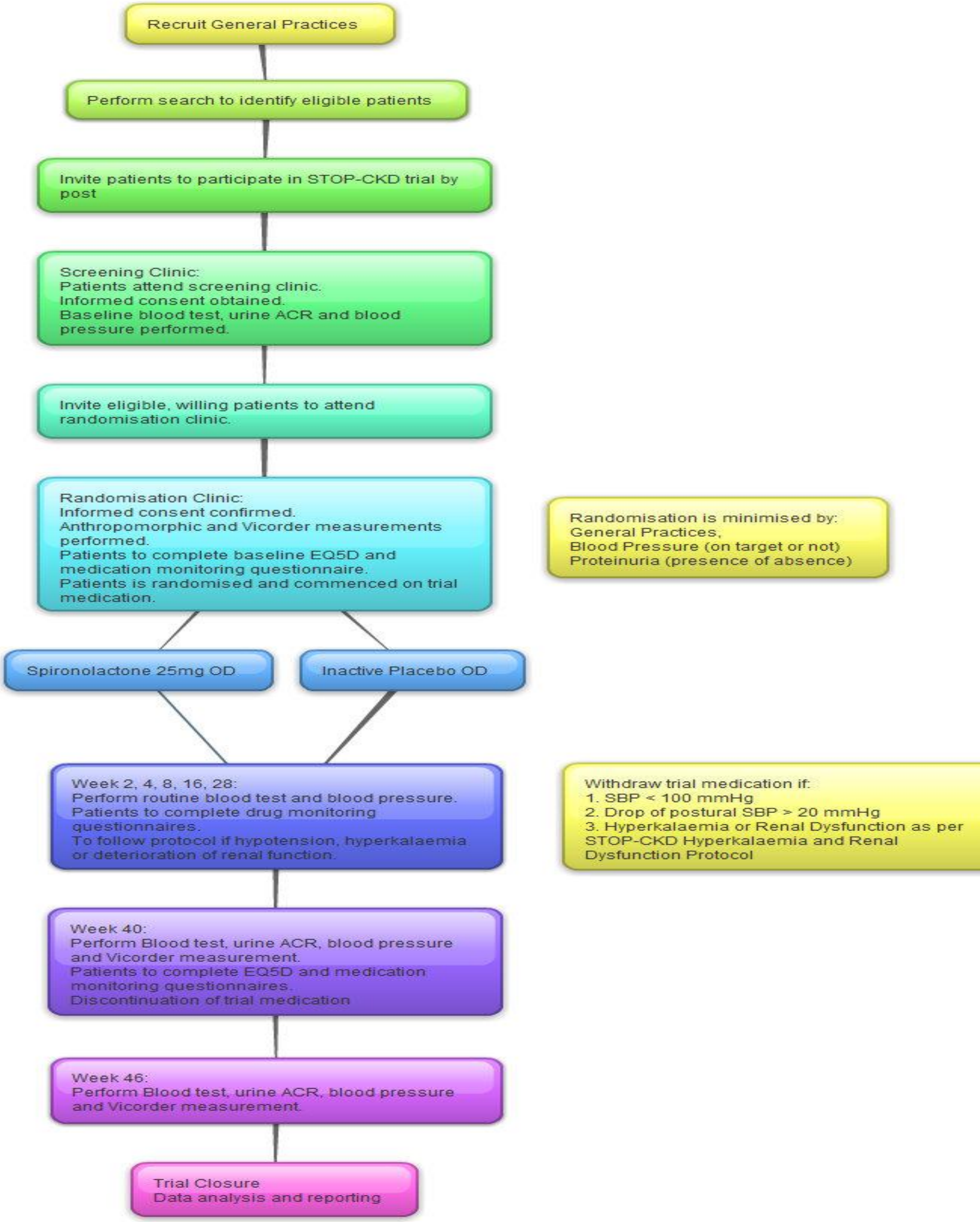

2 INTRODUCTION

2.1 Background

Chronic Kidney disease (CKD) is a common but poorly recognised risk factor for cardiovascular disease in the community. CKD is defined and categorised in terms of estimated glomerular filtration rate (eGFR) and affects more than 10% of the population. In primary care, the largest group (>50%) is CKD stage 3 defined as eGFR 30 to 59 ml/min/1.73m2 [1-4]. This issue is magnified in patients aged 65 or over where up to 35% of these patients will have eGFR of less than 60 ml/min/1.73m^2. CKD prevalence appears to be increasing with a rise from 10 to 13.1% over the last decade in the US [2].

Only 1.3% of patients with CKD stage 3 are likely to require renal replacement therapy after five years but they are far more likely to suffer from a range of adverse cardiovascular events such as stroke or heart failure [5-9]. The risk of cardiovascular disease is between 2 and 4 times that of control populations and even greater in young patients [10-13]. The presence of proteinuria increases this risk a further two- to four-fold [14, 15].

Cardiovascular disease in CKD differs from that of the general population. Traditional risk factors such as cholesterol and blood pressure are poorly predictive of risk while markers of inflammation, anaemia, hypoalbuminaemia and bone mineral disorder are much more important than in the general population [16, 17]. Although CKD patients have more atherosclerotic disease, two further phenotypes exist:

- Left ventricular hypertrophy accompanied by systolic and diastolic dysfunction;
- Arterial wall thickening, stiffening and calcification (arteriosclerosis).

We and others have shown that these abnormalities are present in patients with early (stage 2 and 3) CKD [18, 19] despite good blood pressure control [20]. The importance of increased arterial stiffness in CKD is evidenced by the strong independent association of this parameter with mortality in patients with CKD [21, 22].

Despite the CV risk, there is a paucity of information on how to treat patients with early CKD as most large studies exclude these patients [23]. The only useful evidence is from post hoc or subgroup analysis of patients with CKD often not known to have this at inclusion [24-28].

The Renin-Angiotensin-Aldosterone System (RAAS) is an attractive target for intervention and inhibitors of this system have been used effectively to reduce hypertension and proteinuria in CKD patients [29-31]. In addition, retrospective analyses of the HOPE (Ramipril in high cardiovascular risk patients) and PROGRESS (perindopril protection against recurrent stroke study) suggested that angiotensin converting enzyme (ACE) inhibitors may

be even more effective in reducing cardiovascular risk in patients with evidence of CKD than in individuals with normal renal function [18, 27]. However prolonged use of ACE inhibitors and angiotensin receptor blockers (ARB) can lead to aldosterone 'escape' and their efficacy in non-proteinuric CKD is less certain [29].

Aldosterone is a mineralocorticoid that is a key effector of the RAAS. In vitro, it is implicated in numerous cardiovascular effects including endothelial dysfunction, transmural arterial inflammation, myocardial and vascular hypertrophy and fibrosis independent of blood pressure control [32-35]. In humans high aldosterone levels have been associated with higher left ventricular mass, increased arterial stiffness, reduced exercise capacity and a more rapid decline in renal function. Treatment with MRBs conferred a powerful prognostic benefit in heart failure in the RALES and EPHESUS studies [36, 37] and was confirmed in a meta-analysis [38]. In CKD patients they are associated with significant decreases in proteinuria [39, 40].

We have been investigating the problem of high cardiovascular morbidity and mortality in CKD for the last four years. We have recently completed and published a double blind, randomised placebo controlled study showing that the addition of the MRB, spironolactone to background ACE or ARB therapy in patients with stage 2 and 3 CKD in a specialist setting effectively and safely reduced LV mass and improved parameters of LV systolic and diastolic function measured by echocardiography and magnetic resonance imaging [41]. Arterial stiffness, measured by pulse wave velocity, was also significantly reduced.

A trial testing whether these changes in intermediate end points are translated into gains in terms of reduced cardiovascular events is required and would need to include many patients recruited from a primary care setting where most CKD stage 3 patients are managed. However, our pilot work suggests that many patients with CKD in primary care are not prescribed ACE inhibitors, do not have controlled blood pressure, are almost 20 years older and have less well defined phenotypes than the patients included in our hospital based study. In addition, concerns on spironolactone resulting in hyperkalaemia and worsened renal function amongst the primary and secondary healthcare professionals have also limited the widespread use of spironolactone, especially in the primary care setting. The incidence of hyperkalaemia related to spironolactone was reported to be between 2 to 17.5% [36, 37, 42-44]. This diverse variation is likely to be due to the difference of the age, prevalence of diabetes mellitus, and use of beta-blocker in the studied population. Similarly, the long-term effect of spironolactone on renal function is uncertain, especially in the chronic kidney disease population. Therefore, before undertaking a large and expensive, appropriately powered clinical trial, further feasibility and safety work is required in primary care.

2.2 Rationale for Current Trial

The aim is to carry out preliminary development work for, and to test the feasibility of, a randomised controlled trial (RCT). The ultimate purpose of the RCT is to determine whether cardiovascular events might safely be reduced with the use of spironolactone in addition to standard management for chronic kidney disease (CKD) in a primary care setting.

The questions that need to be addressed are:

1) What are patient and primary care physician's attitudes to spironolactone in CKD in a community setting and what potential barriers exist to its use?

Spironolactone is currently uncommonly used in this population (pilot work suggests that <5% are prescribed it) and given the potential number of patients that would be eligible for treatment should the trial be successful it is important that potential barriers to implementation of a large trial are explored with both patients and physicians.

2) What is the recruitment rate to the trial?

In order to determine the likely cost and feasibility of a definitive trial, we need to know how many patients per practice are likely to be recruited into the trial over a given period of time.

3) Are similar effects in intermediate outcome (pulse wave velocity) as seen in a secondary care population achievable with a more pragmatic design in primary care?

This information will underpin the power calculation for a subsequent substantive trial. Key differences in study design between a community population and previous specialist setting are: Lack of run in period, the concomitant medication prescribed, baseline blood pressure control and the characterisation of patients' phenotypes.

4) What rates of hyperkalaemia are seen with the use of spironolactone in a primary care setting and are there differences in potassium measured either in plasma and serum, sent by standard transport to the laboratory from primary care versus samples centrifuged on site and rapid transport?

Spironolactone is well documented to cause hyperkalaemia and in primary care this is complicated by the fact that spuriously high potassium levels may occur due to delays in transport to the laboratory. Information regarding hyperkalaemia in a community setting is an important feature in the design of the larger trial.

In addition to addressing the above questions, a further aim of this project is to pilot all the procedures that will be used in the proposed definitive trial.

3 TRIAL OBJECTIVES

- The primary objective is to determine the effect of spironolactone on arterial stiffness in CKD Stage 3 population

- The secondary objectives are to:
  - o Determine the safety of spironolactone in CKD stage 3 in primary care setting, in regards to the incidence of hyperkalaemia and worsened renal function.
  - o Examine whether the different methods of serum analysis affect the rate of hyperkalaemia seen in primary care.
  - o Assess the effect of low dose spironolactone on blood pressure and albuminuria in CKD 3.
  - o Examine patients' and healthcare professionals' attitudes towards CKD as well as research in CKD in the community setting.
  - o Explore patients' and healthcare professionals' attitudes towards the use of spironolactone in CKD in a community setting and the potential barriers which may that might exist to its use.

4 TRIAL DESIGN

4.1 Plan of Investigation

This is a randomised controlled trial aiming to examine the effect of spironolactone on arterial stiffness outcome in patients with CKD stage 3 and its safety in the community setting. This study is a double- blinded and placebo- controlled trial involving 240 patients. The study period is 46 weeks. The plan of investigation is as followed:

- Up to ten primary care practices within South Birmingham will be recruited to take part in this study.
- Potential subjects within these practices will be identified by general practitioners by searching computerised primary care clinical records for patients with biochemical evidence of CKD stage 3 (eGFR 30-59ml/min/1.73m^2).
- Invitation to participate in STOP-CKD together with Patient information Sheet (PIS) will be sent out to potentially eligible patients.
- Potentially eligible patients will be invited to attend research 'screening clinic' at their own practice where the trial will be further explained verbally and written information supplied.
- Written consent will be obtained for willing participants.
- Clinical history, examination, blood pressure measurement and blood and urine tests will be performed during the 'screening clinic'.
- Blood test on **'screening clinic'** will confirm the diagnosis of CKD 3: eGFR 30-59 ml/min/1.73m^2 using the MDRD (Modification of Diet in Renal Disease) equation. Urine test (urine ACR) will exclude patients who have ACR > 70mg/mmol.

- Willing and eligible patients will then be invited back no more than two weeks after their initial visit (**screening clinic**) to attend for randomisation clinic (**randomisation clinic**).
- During '**randomisation visit'**, informed consent will be sought again before randomisation to commence on trial medication. They will be given time and opportunity with the research clinician or research nurse to discuss or ask the clinician or the research nurse questions regarding their participation in the study. All questions or concerns about the trial should be answered to the satisfaction of the patient. It should be explained that they are free to refuse to take part and informed about their right to withdraw from the trial at any time. If the patient agrees to take part in the trial they should be asked to sign and date the approved Informed Consent Form, which should also be signed and dated by the Investigator. A copy of the Informed Consent Form should be given to the patient, a copy filed in the hospital notes and a copy filed in the Investigator Site File.
- Patients who agree to be enrolled into the study will have their pulse wave velocity (PWV), pulse wave analysis (PWA) and blood pressure measured. Patients will complete the EQ5D (quality of life survey) and medication monitoring questionnaires. They will be randomised between inactive placebo and spironolactone 25 mg daily for 40 weeks.
- Frequent checks of electrolytes, BP measurement and drug monitoring questionnaire will be undertaken at 2, 4, 8, 16 and 28 weeks after the **randomisation visit**. (See section 6. Study Procedure for details).
- Patients with persistently elevated BP > 150/90 mmHg will be referred to their corresponding general practitioner for blood pressure management as per NICE guidelines.
- Subjects with hyperkalaemia or renal function deterioration on the follow-up blood tests during the study period will be managed according to 'STOP-CKD hyperkalaemia and renal dysfunction management protocol' (see **Appendix A**).
- Subjects with an increment of serum creatinine ≥ 30% or reduction of eGFR ≥ 25% from baseline (screening visit eGFR) will be withdrawn from the trial medication.
- Subject with >20 mmHg systolic postural drop in blood pressure during the trial and/or the systolic blood pressure drops to below 100 mmHg will be withdrawn from the trial medication.
- All subjects will have repeat blood and urine test as well as blood pressure, PWV, PWA measurements performed at 40 weeks after the **randomisation visit.** They will also complete the EQ5D and drug monitoring questionnaires at 40 weeks.
- **All subjects will stop trial medication at 40 week after the randomisation visit.**
- There will be a wash-out period of trial medication for 6 weeks. All subjects will have repeat blood and urine tests as well as blood pressure, PWV and PWA measurements performed again at 46 weeks.

Other aspects of this study:

- **Quality Control of Laboratory tests**
    - We will audit time from blood sampling to analysis of sample in the biochemistry laboratory in the Queen Elizabeth Hospital Birmingham.
    - Up to 100 patients enrolled in this trial will have laboratory tests sent both in standard fashion utilising routine transport of samples as well as being centrifuged on site (recruited GP surgeries) and transport them rapidly to laboratory. We aim to compare the serum potassium results processed via these two methods.

- **Qualitative analysis to address development and feasibility issues**
  The pilot RCT will answer research questions 2-4 and additional qualitative work will focus on research questions:

1. What are patients' and general practitioners' attitudes to CKD?
2. What are patients' and general practitioners' attitudes to research in CKD in a community setting and what potential barriers exist for participation?
3. What are general practitioners' and patients' attitudes to spironolactone in CKD in a community setting and what potential barriers exist to its use?

General Practitioners (GPs) and patients who have been invited to participate in the STOP-CKD randomised control trial will also be invited to participate in the qualitative interview study. GPs and patients will receive information sheet on the interview study. Informed, written consent for interview study will be obtained prior to their participation in this qualitative research study.

This interview study aims to understand GPs' and patients' views on CKD, research in CKD as well as the use of spironolactone in CKD in the community setting. The outcome of this qualitative study will contribute to how trial procedures might be modified for a future definitive trial and improve our understanding of the factors which may limit the prescription of spironolactone in the community settings.

A grounded theory approach will be used to guide sampling, data collection and analysis. Purposive sampling will allow for maximum variety of patient and GP characteristics. Up to 30 patients and 30 GPs will be selected for interview. Patient sampling will ensure representation of views from different ages, ethnicity, socio-economic status and gender. We will aim to include both patients who withdraw from the trial after it has started and patients who participate in the complete trial. GP sampling will ensure representation of different ages of GP, practice location and practice size. Interviewing will continue till data saturation has been achieved.

Data sampling technique includes one to one interview as well as focus group. The interviews will be confidential and face to face using a topic prompt which will be refined over the course of the initial interviews. They will be undertaken by research fellow (KN), supervised by SG. The interviews will be carried out in a place convenient to the interviewees (home or GP surgery).

All interviews will be audio-taped and transcribed verbatim. Transcriptions will be read and checked for accuracy by the researcher and the text entered into a computerised database using the NVivo qualitative software package. Interviewees will be sent a copy of their interview summary, asked whether they agree with it and whether they have any additional comments. Constant comparative analysis will be used to interpret the data. Data collection and analysis will be iterative with new data being used to confirm or challenge the emerging concept.

4.2 Trial Outcome Measures

- Primary end point
    - Change in carotid-femoral pulse wave velocity between baseline and 40 weeks

- Secondary end-points
    - Incidence of hyperkalaemia
    - Change in blood pressure (systolic and diastolic)
    - Change in eGFR
    - Change in ACR
    - Change in pulse wave analysis
    - Tolerability of spironolactone
        - Incidence of hyperkalaemia with serum potassium above 5.5mmol/L
        - Incidence of hyperkalaemia with serum potassium above 6mmol/L
        - Incidence of increment of creatinine by more than 30% or reduction of eGFR by more than 25% from baseline
        - Incidence of other side effects leading to withdrawal of spironolactone
        - Incidence of other adverse reactions or events defined by the Medicines and Healthcare products Regulatory Agency (MHRA)

- Health related quality of life as measured by EQ5D
- Difference in serum potassium reading using two different methods of transport and analysis

- Qualitative data outcome on patients' and primary care physicians' attitudes towards spironolactone in CKD in community setting and the potential barriers exist to its use.
- Qualitative data outcome on patients' and primary care physicians' attitudes to CKD and research in CKD in a community setting and the potential barriers exist for participation.

4.3 Study Timetable

**Trial Task**	**Month:**	1-3	4-6	7-9	10-12	13-15	16-18	19-21	22-24
Ethics, practice recruitment		■							
Practice visits, training of staff and identify eligible patients			■						
Patient recruitment and randomisation, data collection				■	■	■	■		
Data entry and follow up				■	■	■	■		
Data cleaning and verification and analysis								■	
Final write-up									■

5 PARTICIPANT ENTRY

5.1 Pre-Randomisation Evaluations

Potential subjects will be identified by searching computerised primary care clinical records for patients with biochemical evidence of CKD stage 3 (eGFR 30-59 ml/min/1.73m^2). Potentially eligible patients will receive invitation to participate in STOP-CKD study and patient information sheet. They will be invited to attend a baseline clinic at their own practice where the trial will be explained verbally and written information supplied. Written consent will be obtained prior to enrolment into the study.

5.2 Sample Size

We aim to recruit 240 patients to account for a drop-out rate of 20%, which will result in at least 200 patients completing this randomised control trial with 100 patients in each arm (inactive placebo versus spironolactone).

Power calculation: The primary end point on which sample size and power has been calculated is pulse wave velocity (PWV). Using the data from our recent study of the effect of spironolactone, the standard deviation (SD) of the change in PWV was 1.0m/s in the active treatment group and 0.9m/s in the control group. Hence, 100 subjects in each arm will provide 90% power with an alpha value of 0.05 to demonstrate a change in PWV of 0.5m/s.

5.3 Inclusion Criteria

- Age over 18 years
- Diagnosis of CKD Stage 3 (eGFR 30-59 ml/min/1.73m^2) using the MDRD (Modification of Diet in Renal Disease) equation.

5.4 Exclusion Criteria

- Diabetes Mellitus
- Terminal disease or felt otherwise unsuitable by their general practitioner (GP)
- Chronic heart failure i.e. a clinical diagnosis or known ejection fraction (EF) <55%
- Atrial fibrillation
- Alcohol or drug abuse
- Inability to comply with trial medication and follow-up
- Documented previous hyperkalaemia or intolerance of spironolactone
- Documented Addison's disease and/or on fludrocortisone
- Severe hypertension: blood pressure ≥ 180/110 mmHg
- Systolic blood pressure less than 120mmHg
- Recent acute kidney injury or hospital admission (within past 6 weeks)
- Chronic diarrhoea
- Pregnancy
- ACR>70 mg/mmol (will require specialist referral if not already made)
- Serum potassium ≥ 5 mmol/L on screening blood test

- Concomitant co-trimoxazole medication
- Concomitant angiotensin-converting enzyme inhibitor AND angiotensin II receptor blocker medication

5.5 Withdrawal Criteria

Subjects will be withdrawn from the trial if they choose not to continue or if their GPs feel that continued participation in the trial is inappropriate or if they are no longer eligible due to:

a. **Hypotension:**
If there is >20 mmHg systolic postural drop in blood pressure during the trial and/or the systolic blood pressure drops to below 100 mmHg then the trial medication will be discontinued.

b. **Hyperkalaemia:** (see also Appendix A: STOP-CKD hyperkalaemia management protocol)
If the potassium is between 5.5-6.0 mmol/L, patient will have urgent repeat sampling at the GP surgery. If repeat potassium is below 5.5 mmol/L, patient will continue with the trial medication. If the repeat potassium is in fact between 5.5- 6.0 mmol/L, the trial medication will be reduced to alternate days and patient will receive low potassium diet advice (see appendix D). Repeat blood test will be performed after one week.
If the potassium is at or above 6.0 mmol/L, urgent repeat sampling will be performed at QEHB. If the repeat potassium is in fact at or above 6.0 mmol/L, the trial medication will be discontinued immediately.

c. **Deterioration of renal function:** (see also Appendix A: STOP-CKD hyperkalaemia management protocol)
If plasma creatinine increase is ≥ 30% or eGFR decrease is ≥25% from the baseline at any time point, the trial medication will be discontinued and the GP will be advised to refer the patient to specialist care.

d. **Serum sodium < 130 mEq/L on 2 occasions:**
To withdraw trial medication.

e. **Gynaecomastia, impotence, diminished libido in male or hirsutism, oligomenorrhoea, amenorrhoea, menorrhagia, breast tenderness in female:**
To withdraw trial medication if patient is intolerant of the side effect/effects.

f. **Headache or Lethargy:**
To withdraw trial medication if symptom persists for >1 week.

g. **Confusion, ataxia or drowsiness:**
To withdraw trial medication.

h. **Rash, Lichen planus or lupus-like syndrome:**

To withdraw trial medication immediately.

i. **General abdominal discomfort:**
To withdraw trial medication if persistent discomfort for > 1 week.

j. **Diarrhoea or vomiting:**
To withdraw trial medication if symptoms persist for > 3 days.

k. **Gastric/ duodenal ulcer or bleeding:**
To withdraw trial medication.

l. **Agranulocytosis:**
To withdraw trial medication

m. **Hepatitis:**
To withdraw trial medication.

n. **Benign adenoma or thyroid, testes, malignant breast tumours, hepatocellular carcinoma or leukemia:**
To withdraw trial medication.

Full details of the reason(s) for withdrawal should be recorded on the Case Report Forms (CRFs) if healthcare professionals-initiated, otherwise a simple statement reflecting patient preference will suffice. Patients who withdraw from trial treatment but continue with on-going follow-up and data collection should be followed-up in accordance with the protocol.

6 STUDY PROCEDURES

The schedule of assessments and investigations required is described below and summarised in Figure 1. This information should be recorded in the patient notes where not explicitly required in the Case Report Forms.

Screening Visit

- Valid Informed Consent gained.
- Full demographic details including age, self-assigned race and postcode.
- Relevant medical history taken.
- Concomitant medication (description of other medication prescribed for more than 7 days and taken within one month of randomisation).
- Screening blood tests: full blood count; urea + creatinine + electrolytes; liver function tests, bone profile, random serum glucose, pro-thrombotic/fibrotic/inflammatory markers.
- Urinalysis using albumin: creatinine ratio (ACR).

Refer to patients' General Practitioner (GP) if:

- **BP ≥ 180/11mmHg**
- **ACR ≥ 70 mg/mmol**: to refer to GP to consider referral to nephrology specialist if patients have not been reviewed by nephrologist in the past 5 years since the diagnosis.
- **ACR= 30-69 mg/mmol and BP ≥ 140/90 mmHg and NOT on either angiotensin converting enzyme inhibitor (ACEI) or angiotensin receptor blocker (ARB)**: to refer to GP to consider for ACEI/ARB. Patients will be re-invited to participate in STOP-CKD study after they have been on ACEI/ARB for at least 6 weeks.
- **ACR= 30-69 mg/mmol with haematuria**: to refer to GP for review.

Eligible and willing patients will be invited back to Randomisation Visit within 2 weeks after Screening Visit.

Randomisation Visit (Week 0)

- Valid Informed Consent gained for randomisation.
- Relevant medical history taken.
- Medication Monitoring Questionnaire. (*see appendix F*)
- Quality of Life Questionnaire (EQ5D-5L). (*see appendix E*)
- Weight, height and waist/hip ratio measurements.
- Office blood pressure measurement using a validated automated device after 5 minutes rest plus after one minute of standing.
- Pulse wave velocity and pulse wave analysis measured using Vicorder Device with added cardiovascular software. This device is simple to use in a primary care setting and has recently been assessed and validated against the most commonly used applanation tonometry device.
- Patients will then be randomised to treatment with spironolactone 25mg once daily or inactive placebo. (please refer to Section 7: Randomisation for details)

Week 2, 4, 8, 16, 28 after Randomisation visit

- Medication Monitoring Questionnaire.
- Record of compliance to trial medication.
- Office blood pressure measurement using a validated automated device.
- Patients with persistently elevated BP > 150/90 mmHg at both week 2 and week 4 will be referred to their corresponding general practitioner for blood pressure management as per NICE guidelines.
- Blood Sample – full blood count, renal, hepatic profile.
- Hyperkalaemia and deterioration of renal function will be managed according to STOP-CKD hyperkalaemia and renal dysfunction management protocol (Appendix A).
- If there is >20 mmHg systolic postural drop in blood pressure during the trial and/or the systolic blood pressure drops to below 100 mmHg, the trial medication will be discontinued.

Week 40 after Randomisation Clinic

- Medication Monitoring Questionnaire.
- Record Compliance to trial medication.
- Quality of Life Questionnaire: EQ5D-5L.
- Office blood pressure measurement using a validated automated device.
- Pulse wave velocity and pulse wave analysis measured using Vicorder Device with added cardiovascular software.
- Blood Sample – full blood count, renal, hepatic and bone profile as well as pro-thrombotic/fibrotic/inflammatory markers.
- Urinalysis for urine ACR.
- Discontinue all trial medication.
- Patients should not have any change of antihypertensive medication between week 40 and week 46.

Week 46 after Randomisation Clinic

- Office blood pressure measurement using a validated automated device.
- Pulse wave velocity and pulse wave analysis measured using Vicorder Device with added cardiovascular software.
- Blood Sample – full blood count, renal, hepatic and bone profile as well as pro-thrombotic/fibrotic/inflammatory markers.
- Urinalysis for urine ACR.
- Trial closes.

Figure 1: Flowchart of Assessments

This table provides a summary of the trial-related procedures required; refer also to full text in section 4.2

		**Treatment and Follow-up**							
**Visit:**	**Screening**	**Randomisation**	**Week 2**	**Week 4**	**Week 8**	**Week 16**	**Week 28**	**Week 40**	**Week 46**
Valid informed consent gained	X	X							
Full demographic details	X								
Relevant medical history taken	X	X							
Concomitant medications	X								
Weight, Height, Waist/Hip ratio measurements		X							
Blood pressure measurement		X	X	X	X	X	X	X	X
Pulse Wave Velocity and pulse wave analysis measurement		X						X	X
Routine Blood sampling: FBC, renal, hepatic, bone profile	X		X	X	X	X	X	X	X
Pro-thrombotic/fibrotics/Inflammatory Markers	X							X	X
Urine ACR	X							X	X
EQ5D-5L Questionnaire		X						X	
Medication Monitoring Questionnaire		X	X	X	X	X	X	X	X

7 RANDOMISATION AND ENROLMENT PROCEDURE

7.1 Randomisation or Registration Practicalities

All investigators, practice staff and patients will be blinded to treatment. Randomisation will be undertaken using the Primary Care Clinical Research and Trials Unit (PC-CRTU, fully accredited by the NIHR as a trials unit) secured web-based randomisation system. Randomisation assignment will be computer generated and stratified by practice, patient's blood pressure (on target or not on target) and presence of albuminuria. Patients will then be randomised to treatment with spironolactone 25 mg once daily or inactive placebo.

7.2 Unblinding

All investigators will be unblinded after the completion of 46 weeks follow-up of all subjects and closure of the trial. The practice staff and all patients involved in the study, including those who withdraw, will also be informed of their trial medications (spironolactone or inactive placebo).

During the trial, codebreak should be avoided whenever possible. If emergency unblinding is deemed to be appropriate, all STOP-CKD research team members have access to break the randomisation code in emergency situations. When assessing SAEs it should be assumed that the patient received spironolactone (not inactive placebo). If the event is thought to be related, unblinding will be performed by the coordinating centre.

To report serious adverse event/reaction
OR
To break the randomisation code for a patient:

☏ STOP-CKD hotline on:
0 800 9230329

8 TREATMENTS

8.1 Treatment Arms

STOP -CKD is a double-blinded randomised controlled trial involving two treatment arms:

- Spironolactone 25mg orally, once daily.
- Inactive placebo, orally, once daily.

During the trial, subjects may reduce the trial medication to once, every two days according to STOP-CKD hyperkalaemia and renal dysfunction management protocol.

The trial medication will be supplied by Royal Free Hospital and funded via NIHR RfPB grant for this study. The trial medication will be labelled and packaged by Royal Free Hospital prior to delivery to the local Lloyd Pharmacy closest to the recruited general practice for participant to collect.

8.2 Dose Modifications for Toxicity

Information on side effects of spironolactone is derived from British National Formulary [45].

Common Terminology Criteria for Adverse Events (CTCAE) v4.0 is used to classified frequency of undesirable effects of the medication.

Very common: ≥1/10
Common: ≥1/100; < 1/10
Uncommon: ≥ 1/1,000; < 1/100
Rare: ≥ 1/10,000; < 1/1,000
Very rare: < 1/10,000
Unknown: cannot be estimated from the available data

**Metabolic**	Common	Hyperkalaemia [46]	To be managed according to STOP-CKD hyperkalaemia and renal dysfunction protocol (Appendix A)
	Common	Hyponatremia	To withdraw trial medication if serum Na+ <130 mEq/L on 2 occasions
**Renal**	Very common/ common	Renal Dysfunction	To be managed according to STOP-CKD hyperkalaemia and renal dysfunction protocol (Appendix A)
**Endocrine**	Very common/ common	*Male*: Gynaecomastia, impotence, diminished	To withdraw trial medication if patient is intolerant of the

		libido.<br>*Female*: hirsutism, oligomenorrhoea, amenorrhoea, menorrhagia, breast tenderness	side effect/effects.
**Nervous system**	Common	Headache	To withdraw trial medication if symptom persists for >1 week.
	Unknown	Confusion, ataxia, drowsiness<br><br>Lethargy	To rule out electrolyte disturbance leading to symptoms. To withdraw trial medication.<br><br>To withdraw trial medication if symptom persists for > 1 week.
**Dermatologic**	Common	Rash	To withdraw trial medication
	Rare	Lichen planus, lupus-like syndrome	To withdraw trial medication
**Hypersensitivity**	Uncommon	Anaphylaxis, contact dermatitis, eosinophilia	To withdraw trial medication immediately
**Gastrointestinal**	Common	General abdominal discomfort<br><br>Diarrhoea, vomiting	To withdraw trial medication if persistent discomfort for > 1 weeks<br><br>To withdraw trial medication if persistent diarrhoea or vomiting for >3 days.
	Very rare	Gastric/ duodenal ulcer or bleeding	To withdraw trial medication
**Haematologic**	Rare	Agranulocytosis	To withdraw trial medication
**Hepatic**	Rare	Hepatitis	To withdraw trial medication
**Oncologic**	Unknown	Animal studies suggested association between spironolactone with benign adenoma of the thyroid and testes, malignant breast tumours, hepatocellular carcinoma and leukemia.	To withdraw trial medication

8.3 Pre-Medication

Not required

8.4 Interaction with Other Drugs [45, 47, 48]

Angiotensin Converting Enzyme (ACE) inhibitors or Angiotensin II Receptor Blocker (ARB): Concomitant administration of ACE inhibitors and/o

r ARB with potassium-sparing diuretics is associated with increased risk of hyperkalaemia. Serum K^+ and renal function should be **monitored** carefully and on regular basis.

Antihypertensives - potentiation of the effect of antihypertensive drugs occurs and their dosage may need to be reduced when spironolactone is added to the treatment regime, and then adjusted as necessary.

Anti-diabetics: Administration with chlorpropamide may increase risk of hyponatraemia.

Alcohol, barbiturates, or narcotics: Potentiation of orthostatic hypotension may occur.

Aspirin: May reduce the diuretic effect of spironolactone.

Ciclosporin: Co-administration of potassium-sparing diuretics with ciclosporin may result in hyperkalaemia. Avoid concurrent use of spironolactone and ciclosporin. If concurrent therapy is necessary, monitor serum potassium levels for persistent elevations in patients.

Corticosteroids, ACTH: Intensified electrolyte depletion, particularly hypokalemia, may occur.

Coumarins: In patients receiving oral anticoagulant therapy with warfarin, the prothrombin time ratio or INR (international normalised ratio) should be monitored with the addition and withdrawal of treatment with spironolactone, and should be reassessed periodically during concurrent therapy. Adjustments of the warfarin dose may be necessary in order to maintain the desired level of anticoagulation.

Digoxin: Spironolactone has been shown to increase the half-life of digoxin. This may result in increased serum digoxin levels and subsequent digitalis toxicity. It may be necessary to reduce the maintenance and digitalization doses when Spironolactone is administered, and the patient should be carefully **monitored** to avoid over or under-digitalization.

Drug/Laboratory test interactions: Several reports of possible interference with digoxin radioimmunoassay by Spironolactone, or its metabolites, have appeared in the literature. Neither the extent nor the potential clinical significance of its interference (which may be assay-specific) has been fully established.

Diuretics: Spironolactone should not be administered concurrently with other potassium- sparing diuretics as this may induce hyperkalaemia. Potassium canrenoate, a metabolite of spironolactone, has been shown to cause myeloid leukaemia in rats.

Lithium: Concurrent use of lithium and spironolactone may result in increased lithium concentrations and lithium toxicity (weakness, tremor, excessive thirst, and confusion) due to decreased lithium excretion. If concomitant therapy is necessary monitor serum lithium levels within the first five to seven days of adding or discontinuing spironolactone and periodically thereafter. Lower lithium doses may be required with concomitant spironolactone therapy.

Nonsteroidal anti-inflammatory drugs (NSAIDs): Combination of NSAIDs, e.g., indomethacin, with potassium-sparing diuretics has been associated with severe hyperkalemia and should be **avoided** in CKD Stage 3 population.

Potassium salts: Potassium supplements are contraindicated except in cases of initial potassium depletion. If potassium supplementation is considered essential, serum electrolytes should be monitored.

Pregnancy: Teratogenic effects. Pregnancy Category C.

Sympathomimetics: Spironolactone reduces vascular responsiveness to noradrenaline **(**norepinephrine**)**; caution should be exercised in the management of patients subjected to regional or general anaesthesia.

Tacrolimus: Spironolactone should not be used in patients undergoing therapy with tacrolimus as concomitant use has resulted in mild to severe hyperkalaemia.

Trimethoprim-sulfamethoxazole: Among older patients receiving spironolactone, treatment with trimethoprim-sulfamethoxazole was associated with a major increase in the risk of admission to hospital for hyperkalaemia. This drug combination should be avoided when possible [48].

Ulcer healing drugs: As carbenoxolone may cause sodium retention and thus decrease the effectiveness of spironolactone, concurrent use of the two agents should be avoided.

8.5 Dispensing and Accountability

As STOP-CKD is a double-blinded randomised controlled trial, FP10 prescriptions are therefore not suitable. Royal Free Hospital will manufacture and package the drug and placebo. These trial packs will then be delivered to local Lloyd pharmacy closest to the recruited general practice and dispensed from there.

It is very important that accurate drug dispensing, schedule and compliance data are recorded. The trial will therefore utilise the expertise of a named Lloyd pharmacist to assist with completion of such records.

Accurate collection of this data will not only support toxicity and quality of life evaluations being conducted as part of the study but may be pivotal to the overall success of this trial. Any problems relating to pharmacy issues should be addressed to the Study Pharmacy Advisor.

9 TRIAL MANAGEMENT

STOP-CKD will be coordinated by the Primary Care Clinical Research and Trials Unit (PC-CRTU, fully accredited by NIHR as a trials unit) at the University of Birmingham according to the current guidelines for Good Clinical Practice. Conduction of the study may be monitored by research team to confirm compliance with the protocol and the protection of patients' rights as detailed in the Declaration of Helsinki.

9.1 Roles and Responsibilities

The CI takes overall responsibility for the conduct of study. Any delegated or devolved responsibility will be documented on the delegation log. It is the CI's responsibility to ensure that staffs are appropriately trained to perform the tasks that they are delegated to and that it is appropriately documented on the delegation log. (See *Appendix H* for the roles and responsibilities of trial staff).

9.2 Trial Steering Committee

A Trial Steering Committee (TSC) will be appointed and will provide the overall supervision for the trial, in particular: trial progress, protocol compliance, patient safety and review of updated information.

The TSC will include the Trial Management Group (namely the CI, co-investigators, study manager, and statistician), two lay representatives (Paul Cornick and Nick Flint) and Dr Robert Cramb, Consultant Biochemist who provide expertise in the collection and analysis of laboratory samples and an independent Consultant Nephrologist. The TSC will meet 3-6 monthly depending on the phase of the study. An investigators group will meet monthly to provide oversight of the developing trial with more frequent operational meeting of the CI, trial manager and trial team as required.

9.3 Data Monitoring Committee

An independent Data Monitoring Committee for the trial will be responsible for the regular monitoring of trial data. The committee will consist of a clinician not entering patients into the trial, an academic general practitioner and an independent statistician who will also chair the committee.

The DMC will assess the progress of the trial and give advice on whether the accumulated data from the trial, together with the results from other relevant trials, justifies the continuing recruitment of further patients. The committee will meet in person or by teleconference prior to the trial commencing and then three and six months after initiation of trial. The DMC will make confidential recommendations to the TSC as the decision-making Committee for the trial.

10 PHARMACOVIGILANCE

10.1 Definitions

Adverse Event (AE): any untoward medical occurrence in a patient or clinical trial subject administered a medical product, and which does not necessarily have a causal relationship with the treatment. *An AE can therefore be any unfavourable and unintended sign (including an abnormal laboratory finding), symptom or disease temporarily associated with use of an investigational medicinal product (IMP), whether or not considered related to the IMP.*

Adverse Reaction (AR): all untoward and unintended responses to an IMP, related to any dose administered. *All AEs judged by the reporting investigator or sponsor as having a reasonable causal relationship to an IMP qualify as adverse reactions. The expression 'reasonable causal relationship' means to convey in general that there is evidence or argument to suggest a causal relationship.*

Unexpected Adverse Reaction: an AR, the nature or severity of which is not consistent with the applicable product information (eg. Investigator's Brochure for an unapproved investigational product, or a Summary of Product Characteristics (SmPC) for an authorised product). *When the outcome of the adverse reaction is not consistent with the applicable product information, this adverse reaction should be considered as unexpected. Side effects documented in the SmPC which occur in a more severe form than anticipated are also considered to be unexpected.*

Serious Adverse Event (SAE) or **Serious Adverse Reaction (SAR)**: any untoward medical occurrence or effect that at any dose:

- Results in death
- Is life-threatening – *an event in which the subject was at risk of death at the time of event, not an event that hypothetically might have caused death if it were more severe*
- Requires hospitalisation, or prolongation of existing inpatients' hospitalisation
- Results in persistent or significant disability or incapacity
- Is a congenital anomaly or birth defect

Medical judgement should be exercised in deciding whether an AE/AR is serious in other situations. Important AE/ARs that are not immediately life-threatening or do not result in death or hospitalisation but may jeopardise the subject or require intervention to prevent one of the other outcomes listed above, should also be considered serious.

Suspected Unexpected Serious Adverse Reaction (SUSAR): any suspected adverse reaction related to an IMP that is both unexpected and serious.

10.2 Causality

Most AE/ARs that occur in this trial, whether they are serious or not, will be expected treatment-related toxicities due to the drugs used in this trial. The assignment of the causality should be made by the Investigator responsible for the care of the participant using the definitions in the table below.

If any doubt about the causality exists, the Local Investigator should inform the Study Co-ordination Centre, who will notify the Chief Investigator. The pharmaceutical companies and/or other clinicians may be asked to advice in some cases.

In the case of discrepant views on causality between the Investigator and others, all parties will discuss the case. In the event that no agreement is made, the MHRA will be informed of both points of view.

Relationship	Description
**Unrelated**	There is no evidence of any causal relationship.
**Unlikely**	There is little evidence to suggest a causal relationship (eg. the event did not occur within a reasonable time after administration of the trial medication). However, the influence of other factors may have contributed to the event (eg. the participant's clinical condition, other concomitant treatments).
**Possible**	There is some evidence to suggest a causal relationship (eg. because the event occurs within a reasonable time after administration of the trial medication). However, the influence of other factors may have contributed to the event (eg. the participant's clinical condition, other concomitant treatments).
**Probable**	There is evidence to suggest a causal relationship and the influence of other factors is unlikely.
**Definitely**	There is clear evidence to suggest a causal relationship and other possible contributing factors can be ruled out.
**Not assessable**	There is insufficient or incomplete evidence to make a clinical judgement of the causal relationship.

10.3 Reporting Procedures

All AEs should be reported. Depending on the nature of the event, the reporting procedures below should be followed. Any questions concerning AE reporting should be directed to the Chief Investigator, in the first instance. A flowchart is provided (*Appendix J*) to aid in the reporting procedures.

10.3.1 Non-Serious AE/ARs

All such events, whether expected or not, should be recorded in the toxicity section of the relevant Case Report Form and sent to the Study Co-ordination Centre within one month of the form being due.

10.3.2 SAEs and SARs

An SAE form (QCD **09-04**) should be completed and faxed to the Study Co-ordination Centre for all SAEs and SARs immediately. However, hospitalisations for elective treatment of pre-existing condition do not need reporting as SAEs.

Fatal or life-threatening SAEs and SARs should be reported on the day that the local site is aware of the event, by means of the SAE form. Any additional information relating to the event should be sent within 5 days if the reaction has not resolved at the time of reporting.

10.3.3 SUSARs

Complete the SAE form and send it immediately (preferably by fax), signed and dated to the Study Co-ordination Centre, together with relevant treatment forms and anonymised copies of all relevant investigations.

OR

Contact the Study Co-ordination Centre by telephone and then send the completed SAE form to the Study Co-ordination Centre immediately, as above.

The Study Co-ordination Centre will notify the MHRA and main REC of any SUSARs occurring during the study (fatal and life-threatening SUSARs within 7 days of notification, and non-life-threatening within 15 days). All investigators will be informed of all SUSARs occurring throughout the study.

Local investigators should report any SUSARs and/or SAEs/SARs as required by their Local Research Ethics Committee and/or R&D Office.

Contact Details for Reporting SAEs/SARs and SUSARs

Fax **0121 414 3050,** attention of **Dr Charles Ferro**

11 ASSESSMENT AND FOLLOW-UP

Subjects will be receiving blinded treatment (inactive placebo or spironolactone) for 40 weeks and followed-up for 46 weeks after randomisation. (*Please refer to Section 6: Study Procedure for the details of each assessment*)

11.1 Loss to Follow-Up

We plan to recruit an extra 20 patients into each treatment arm in the hope that this will allow for any participants that are lost to follow-up.

If participants miss week 2 or week 4 trial follow-up, we will inform the practice, contact the patients and re-invite the participants to the next follow-up clinic within a week.

If participants missed week 8, 16, 28, 40 or 46 trial follow-up, we will inform the practice, contact the patients and re-invite the participants to the next follow-up clinic within 2 weeks.

If participants fail to attend clinic on 2 occasions, we will aim to establish the reason of failure to attend. If participants are deemed not suitable to continue with the study or decide to withdraw from the study, we will withdraw participants from the study and ensure participants return the rest of the unused trial medications.

11.2 Trial Closure

For the purposes of Clinical Trial Authorisation (CTA) under the European Union Directive 2001/20/EC, the study is deemed to have ended 30 days after the last patient undergoes the final assessment at 46 weeks.

For the purposes of Multicentre Research Ethics Committee approval, the study end date is deemed to be the date of last data capture.

12 STATISTICS AND DATA ANALYSIS

12.1 Statistical Plan

The statistical plan has been developed through advice of Prof Nick Freemantle. He will also supervise statistical analysis of this trial.

Power Calculation: The primary end point on which sample size and power has been calculated is PWV. Using the data from our recent study of the effect of spironolactone the SD of the change in PWV was 1.0 m/s in the active treatment group and 0.9 m/s in the control group. Thus, 100 subjects per group will provide 90% power at with an alpha value of 0.05 to demonstrate a change in PWV of 0.5 m/s. We will recruit 240 patients to account for a drop-out rate of 20%.

12.2 Analysis Plan

Difference in the primary outcome between experimental conditions will be assessed using generalised mixed models. Repeated measures within a subject will be characterised as R side residual overdispersion parameters, accounting for baseline values. Frequency differences will be tested by Fisher's Exact test. A pre-specified multivariable analysis will examine the influence of change in systolic BP and other relevant factors on changes in the primary end point.

12.3 Data Handling, Record Keeping and Retention

To enable monitoring, peer review and/or audits from Health Authorities, the Investigator must agree to keep records, including the identity of all participating subjects (sufficient information to link records e.g. CRFs and GP notes), all original signed Informed Consent Forms, copies of all CRFs and detailed records of drug disposition.

To comply with international regulations these records should be retained by the Investigator for at least 5 years.

12.4 Data Access and Quality Assurance

The STOP-CKD database system will be located within PC-CRTU.

The security of the System is governed by the following relevant policies of the University of Birmingham:

- Data Protection Policy
- Conditions of Use of Computing and Network Facilities (version 2.01 17/12/07)
- Information Security Policy of the University of Birmingham (version 1.01 17/12/07)

- Code of Practice on Access to Computing Facilities (version 1.0 15/12/08) – restricted document
- BS7799 compliance
- ISO 27001/2 compliance

The University's Data Protection Policy and the Conditions of Use of Computing and Network Facilities set out the security arrangements under which sensitive data should be processed and stored. The University has designated Mrs Carolyn Pike (Director of Legal Services) to act as Data Controller and as Data Protection Officer. Any queries should be directed to the Information Compliance Manager Mrs D Jeynes on 0121 414 3196. The University of Birmingham maintains an Information Security Policy which has specifically been designed to be compliant with the ISO 27001/2 standards, and references the existing Conditions of Use of Computing and Network Facilities and New Codes of Practice encompassing use of computers, data use and security. These policies have been approved by Council, the University's governing body, and have been implemented across the whole University. The Study Centre has arrangements in place for the secure storage and processing of the study data which comply with the above policies.

The System shall incorporate the following security countermeasures:

<u>Physical security measures:</u>
Access to the Study Centre is restricted to members of the department using a swipe card system. Any repairs to the System will be carried out on-site and under supervision. A burglar alarm is in operation at times when no members of staff are present. Full backups take place daily and tapes/disks are stored in a fire-proof safe.

<u>Logical measures for access control and privilege management:</u>
The System will only be accessible to persons authorised by the CI. Electronic data are stored on access controlled servers. New requests for access to the system, e.g. for newly appointed staff, have to be approved by the Trial Manager. Patient identifiable data are stored separately from non-identifiable data when they are no longer required for linkage or quality checking. All members of the research team are trained in the appropriate use of confidential information. The data received will be separately archived after personal data has been deleted. The CI will have responsibility for access to the archived material.

All staff members are required to sign an undertaking of confidentiality which if broken would be likely to lead to dismissal.

Network security measures:
The University of Birmingham maintains a 'front door' site firewall to protect the network from external threats. Antivirus software is used on all servers and desktops. The database application and server are hosted on a separate secure network protected by a further hardware firewall. This network can only be accessed via our Citrix Access Gateway SSL VPN appliances.

System Management
The System shall be developed by the PC-CRTU Programming Team.

The System shall be implemented and maintained by the PC-CRTU Programming Team in conjunction with the IT Manager who is responsible for the implementation of security configurations and the CI. In case of disposal or replacement of the System any media that may contain patient identifiable data will be securely wiped. Equipment will then be physically destroyed by a University of Birmingham approved contractor. The System will have a three year standard limited warranty with on-site service.

System Design
The System shall comprise of a database and a data entry application.

Data entry is via a Windows application hosted on a PC-CRTU database. The database server is only accessible from within the university network and the firewall is configured to allow access from specific machines only. Administrative access is restricted to PC-CRTU but some read only access can be enabled for study staff based at the Study Centre. SQL server allows us to specify access at a table level to view, add, edit and delete. This acts as additional security on top of that enforced by the application.

The application server is accessible over the internet via PC-CRTU database. All traffic to and from the application is automatically encrypted using SSL (128 bit). All users are required to log on to the system using a username and strong password. The application and the database itself use role based security controls. Users can only edit and view data that they have been given access to (via their role).

Operational Processes
The patient identifiable/sensitive data will be collected from a combination of sources including directly from participants (onto paper), from medical records (onto paper or secure database). All identifiable data will be collected following patient consent. Paper records will be kept securely in locked filing cabinets within secure areas.

The data will be processed and stored within the Study Centre (University of Birmingham), in accordance the Conditions of Use of Computing and Network Facilities (version 2.01 Dec 2007), the Information Security Policy of the University of Birmingham (version 1.01 dated 17th December 2007).

Processing of all patient identifiable data received for this part of the study takes place in the Study Centre. For back-up, encrypted copies of the data are stored in a secure area of the departmental file server and are backed up tape nightly. Access is limited to authorised study staff and the PC-CRTU IT Manager.

Patient data are stored in secure SQL server database. Data entry is facilitated by a secure application designed and developed by the PC-CRTU programming team in conjunction with the Trial Manager.

We will allocate study identity numbers for the records of all patients enrolled within this project. Patient identifiable data will be kept confidentially and can be separated from the rest of the data.

Data processing

Statisticians will only have access to anonymised data.

Staffs handling of patient identifiable/sensitive data are trained by the CI, in order to ensure that they comply with university policy. See Section 5.3.4 of University of Birmingham Information Security Policy Document (version 1.01 17 December 2007): All employees are required to be made aware of the risk of breaching confidentiality associated with the copying (including photocopying or other duplication) of confidential or sensitive documents. Authorisation for copying such documents should be obtained from the document owner where documents are classified as highly confidential or above.

Disposal

When the System or its data has completed its purpose/has become redundant or is no longer needed, the following methods will be adopted to dispose of equipment, back-up media or other stored data:

Commercially available data shredder software will be used to electronically shred the file and any external hard drives used for back-up. Any DVDs containing data will be destroyed by physically shredding the DVD.

Any study paper data will be retained in a locked room/filing cabinet within the study centre for a minimum of 15 years after the end of the study for research governance purposes, or securely archived. All paper records will be shredded or securely disposed of at this point.

System Audit

The System shall benefit from the following internal/external audit arrangements:

Internal audit of the system can be carried out at any time by the PC-CRTU, an expert central facility for co-ordinating community based studies. Day-to-day monitoring and assurance of the security of the system is the responsibility of the CI, Trial Manager and IT Team Leader.

An annual IT risk assessment is conducted within Primary Care Clinical Sciences. This is conducted by the departments IT manager. The aim of the risk assessment is to ensure identification and evaluation of risks, and to regularly reassess strategies to deal with any possible threats to the security of the System. The scope of the audit includes, but is not limited to, assessing the design and operation of controls to ensure:

- Necessary policies and procedures are in place
- Access control i.e. network and system security is appropriately implemented, restricted and reviewed to safeguard unauthorised access to or modification of programs and data
- Procedures are in place for secure disposal or destruction of storage media when no longer required.
- Security of data
- Data is retained in accordance with laws and university policies to enable retrieval when needed.

<u>System Protection</u>
The System shall benefit from the following resilience/contingency/disaster recovery arrangements:

The databases are all backed up incrementally hourly with a full backup once a day. This allows us to do a 'point in time restore' if required. This means that we can revert to a previous version of the database if needed. The daily backup is encrypted and then copied to the university file server where it is then backed up to tape. Tapes are retained for 20 weeks in a fire proof safe, after which they are reused for subsequent backups. They are then physically destroyed at the end of their useful life.

Both application and database servers also have a disk image taken regularly meaning that if the hardware was to fail then we could restore the servers as they were at that time very quickly.

In anticipation of hardware failures the servers use multiple hard disks in RAID configuration meaning that if one was to fail then no data would be lost. The servers also have dual power units and are protected from power failures and surges by a UPS. The servers are under warranty with next business day support for repairs.

In the event of serious disruption, total system failure, or major incident the System can be restored using the files from the tapes.

In the event of a security or confidentiality breach both the University's Legal Services and the IT Security Manager (IT Services) will be informed so that it can be investigated if appropriate.

Please also refer to Conditions of Use of Computing and Network Facilities (version 2.01 17/12/07) - Section 9.9 Access to Data:

Anyone who wilfully and knowingly acts to impede a security, disciplinary or operational investigation commits a disciplinary offence. This includes the removal or destruction of relevant data or hardware and the withholding of passwords and encryption keys.

System Level Security Policy Ownership
This SLSP shall be the responsibility of CI, Chief Investigator and shall be reviewed on an annual basis.

Data Protection Registration
The University of Birmingham has Data Protection Registration to cover the purposes of analysis and for the classes of data requested. The University's Data Protection Registration number is Z6195856.

12.5 Case Report Forms (CRFs)

The CRFs must be completed and signed/dated by the Investigator or one of their authorised staff members as soon as the required information is available. The completed originals should be sent to the STOP-CKD Study Office, with a copy held by the Investigator at site. In all cases it remains the responsibility of the Investigator to ensure that they have been completed correctly and that the data are accurate. Entries should be made in ballpoint pen preferably in black ink and must be legible. Any errors should be crossed out and with a single stroke, the correction inserted and the change initialled and dated. If it is not clear why a change has been made, an explanation should be written next to the change. Typing correction fluid should not be used. Data reported on each CRF should be consistent with the source data or the discrepancies should be explained. All sections are to be completed before returning to the STOP-CKD Study Office. If information is not known, this must be clearly indicated by entering NK on the form. All missing and ambiguous data will be queried.

Trial CRFs may be amended as appropriate; this will not constitute a protocol amendment. Revised CRFs should be used by all participating sites with immediate effect.

Case report forms will include:

- Screening Visit Form
- Randomisation Visit Form
- Week 2, 4, 8, 16, 28 Visit Forms
- Week 40 Visit Form
- Week 46 Visit Form
- Withdrawal form
- Adverse Event (AE)/Serious Adverse Event (SAE) Form

13 MONITORING

13.1 Risk Assessment

The study has undergone Peer Review by the National Institute for Health Research.

We have previously completed Chronic Renal Impairment in Birmingham II (CRIB II) study, which was a single-centre (secondary care setting), prospective, double-blind, placebo-controlled, randomised interventional trial examining the effect of spironolactone on left ventricular function and arterial stiffness in 112 patients with CKD Stage 2 or Stage 3, who were already receiving an angiotensin converting enzyme (ACE) inhibitor and/or angiotensin receptor blocker (ARB) for at least 6 months [41]. STOP-CKD study employs similar methodology and safety monitoring as CRIB II, except that STOP-CKD study population is confined to CKD Stage 3 and managed in the primary care setting.

In CRIB II, there was a 4-week open-label run-in phase of 25mg of spironolactone once daily, after which patients were randomised to continue a further 36 weeks of treatment with either 25mg of spironolactone or to receive placebo. During the open-label run-in phase, CRIB II reported 1 case of serious hyperkalaemia (potassium 6.5 mmol/L), 1 patient with hypotension and acute deterioration in renal function (eGFR decreased from 31 to 24 ml/min/1.73m^2). Spironolactone was withdrawn from all the above 3 patients. 6 (5%) patients had potassium levels between 5.5 and 5.9 mmol/L requiring reduction of spironolactone dosage. During blinded treatment between week 4 and 40, 4 patients had potassium levels between 5.5 and 5.9 mmol/L that required a dose reduction to alternate day treatment. Two of these 4 patients were found to have been on placebo after unblinding. No patients were withdrawn due to hyperkalaemia after randomisation, and there were no reported side effects, including gynaecomastia [41].

CRIB II study evidently demonstrated relatively few adverse events associated with the use low dose spironolactone 25mg once daily in CKD Stage 2 and Stage 3 patients in the secondary care setting, provided there was careful monitoring plan in place. Hence, by using the same dose of spironolactone and identical adverse reaction/events monitoring regime, this pilot trial will provide crucial information to ascertain if similar safety profile of spironolactone 25mg once daily can be achieved in the primary care setting in CKD Stage 3 patients.

13.2 Monitoring At Study Co-ordination Centre

The study coordinator will be in regular contact with the recruited practice staffs, research nurse and trial administrator (by phone or email) to check on progress and answer any queries that they may have. Trial staff will check

incoming CRFs for compliance with the protocol, consistent data, missing data and timing.

Research team have engaged with quality assurance at PC-CRTU at consultancy level. Research team will be advised in terms of monitoring plan for the study. Monitoring will be conducted both centrally and on-site while research team visits the general practices.

13.3 Monitoring At Local Site

Monitoring will be done according to the PC-CRTU policy and the STOP-CKD Monitoring Plan. Investigators will allow the trial monitors access to source documents as requested. If a monitoring visit is required, the STOP-CKD Study Office will contact the site to arrange a date for the proposed visit. Data to be verified will include:

- Informed Consent
- Eligibility
- Adverse Events

Any major problems identified during monitoring will be reported to the STOP-CKD Steering Committee. All records will be maintained in accordance with local regulations and in a manner that ensures security and confidentiality. The completed original CRFs are the sole property of the STOP-CKD Steering Committee and should not be made available in any form to third parties (except for authorised representatives of appropriate Health/Regulatory Authorities) without written permission from the STOP-CKD Steering Committee.

14 REGULATORY ISSUES

14.1 Clinical Trial Authorisation (CTA)

This study has Clinical Trial Authorisation from the UK competent authority, MHRA: reference: 21761/0274/001-0001

14.2 Ethics Approval

The Study Co-ordination Centre has obtained approval from the West Midlands- Coventry and Warwickshire Research Ethics Committee. The trial must be submitted for Site Specific Assessment (SSA) at each participating NHS Trust. The Chief Investigator will require a copy of the SSA approval letter before accepting participants into the trial. The trial will be conducted in accordance with the recommendations for physicians involved in research on human subjects adopted by the 18th World Medical Assembly, Helsinki 1964 and later revisions.

14.3 Patient Consent

It is the responsibility of the research clinician or research nurse to obtain fully Informed Written Consent from each patient prior to evaluation of the patient's suitability for the trial as well as enrolment into the trial.

Suitable volunteers will be identified from their medical records by their general practitioner to be invited to attend the Health Centre. The approved Patient Information Sheet will be send out to the identified patients with the invitation to attend the first research clinic ('screening visit'). The volunteers will be given ample time to read the information sheet at home, given time to discuss their participation with others outside of the clinical trials team in order for the patient to reach a decision. During the 'screening visit', volunteers will have the opportunity to discuss about the risks and benefits of participation in the study and chance to ask any question. Informed consent will be sought by the Clinical Staff (Medical or Nursing) and where obtained, will be followed by initial screening blood samples.

Volunteers who are suitable for entry into the study based on the evaluation of the screening visit will then be invited back to the Health Centre within 2 weeks of the initial visit, whereby informed consent will be sought again before randomisation to commence on trial medication. They will be given time and opportunity with the research clinician or research nurse to discuss or ask the clinician or the research nurse questions regarding their participation in the study. All questions or concerns about the trial should be answered to the satisfaction of the patient. It should be explained that they are free to refuse to take part and informed about their right to withdraw from the trial at any time. If the patient agrees to take part in the trial they should be asked to sign and date the approved Informed Consent Form, which should also be signed and

dated by the Investigator. A copy of the Informed Consent Form should be given to the patient, a copy filed in the hospital notes and a copy filed in the Investigator Site File.

Throughout the study the patient will have the opportunity to ask questions about the trial and any new information that may be relevant to the patient's willingness to continue participation in the trial should be shared with the patient in a timely manner.

A copy of the Informed Consent Form and Patient Information Sheet is included in Appendices (Appendix B and Appendix C).

14.4 Confidentiality

Participants' identification data will be required for the registration process. The Study Co-ordination Centre will preserve the confidentiality of participants taking part in the trial, and is registered under the Data Protection Act. Confidentiality will be monitored and consent will be sought from patients for members of the study team, regulatory authorities and the PCT to have direct access to patient medical records. If data is to be transferred outside of the EU, consent must also be sought for this.

14.5 Indemnity

University of Birmingham holds Public Liability (negligent harm) and Clinical Trial (negligent harm) insurance policies, which apply to this trial.

14.6 Sponsor

University of Birmingham will act as the main sponsor for this trial.

14.7 Funding

The National Institute for Health Research, Research for Patient Benefit Scheme is funding this trial.

14.8 Audits

The trial may be subject to inspection and audit by MHRA and University of Birmingham under their remit as sponsor, the Study Co-ordination Centre and other regulatory bodies, to ensure adherence to GCP.

15 FINANCIAL ARRANGEMENTS

15.1 Participant Payments

Participants will be reimbursed for travel expenses where required if requested

15.2 GP Payments

GP practices will be reimbursed for work done in supporting the trial via service support costs.

16 PUBLICATION

The overall question being addressed by this study – is a randomised controlled trial of spironolactone in CKD the community feasible and likely to result in similar effects to those seen in hospital settings? – will be of relevance to the renal research community and the primary care research community. If the answer is yes, a trial is feasible, then we will be actively disseminating the results to general practices and renal colleagues nationwide with the aim of recruiting them to the definitive trial. An obvious mechanism to do this is via the renal speciality group of the Comprehensive Research Network and the Primary Care Research Network. This will be supplemented by meetings that we will organise to explain the trial to interested renal specialists and GPs in their catchment areas. If the answer is no, a trial is not feasible, then the lessons learnt will remain of considerable interest to the research community, and we would present our findings to both a primary care audience (e.g at scientific meetings of the Society for Academic Primary Care and the Confederation of Primary Care Research Organisations) and a renal audience (e.g. at the Renal Association and American Society of Nephrology).

We would anticipate publishing the findings in peer-reviewed journals on the overall results of the pilot study. We will disseminate our results to all participants and via our website and local media plus through patient groups (see above).

All publications and presentations relating to the trial will be authorised by the Trial Steering Committee. The first publication of the trial results will be in the name of Trial Steering Committee, if this does not conflict with the journal's policy. If there are named authors, these will include at least the Chief Investigator, Statistician and Trial Co-ordinator. Members of the Trial Steering Committee and Data Monitoring Committee will be listed and contributors will be cited by name if published in a journal where this does not conflict with the journal's policy. Authorship of parallel studies initiated outside of the Trial Steering Committee will be according to the individuals involved in the project, but must acknowledge the contribution of the Trial Steering Committee and Study Co-ordination Centre.

17 REFERENCES

1. Levey, A.S., et al., *A more accurate method to estimate glomerular filtration rate from serum creatinine: a new prediction equation. Modification of Diet in Renal Disease Study Group.* Annals of internal medicine, 1999. **130**(6): p. 461-70.
2. Coresh, J., et al., *Prevalence of chronic kidney disease and decreased kidney function in the adult US population: Third National Health and Nutrition Examination Survey.* American journal of kidney diseases : the official journal of the National Kidney Foundation, 2003. **41**(1): p. 1-12.
3. Stevens, P.E., et al., *Chronic kidney disease management in the United Kingdom: NEOERICA project results.* Kidney international, 2007. **72**(1): p. 92-9.
4. Hallan, S.I., et al., *Screening strategies for chronic kidney disease in the general population: follow-up of cross sectional health survey.* BMJ, 2006. **333**(7577): p. 1047.
5. Anavekar, N.S., et al., *Relation between renal dysfunction and cardiovascular outcomes after myocardial infarction.* The New England journal of medicine, 2004. **351**(13): p. 1285-95.
6. Abramson, J.L., et al., *Chronic kidney disease, anemia, and incident stroke in a middle-aged, community-based population: the ARIC Study.* Kidney international, 2003. **64**(2): p. 610-5.
7. Glynn, L.G., et al., *Chronic kidney disease and mortality and morbidity among patients with established cardiovascular disease: a West of Ireland community-based cohort study.* Nephrology, dialysis, transplantation : official publication of the European Dialysis and Transplant Association - European Renal Association, 2007. **22**(9): p. 2586-94.
8. Yahalom, G., et al., *Chronic kidney disease and clinical outcome in patients with acute stroke.* Stroke; a journal of cerebral circulation, 2009. **40**(4): p. 1296-303.
9. Keith, D.S., et al., *Longitudinal follow-up and outcomes among a population with chronic kidney disease in a large managed care organization.* Archives of internal medicine, 2004. **164**(6): p. 659-63.
10. van Kuijk, J.P., et al., *The prevalence and prognostic implications of polyvascular atherosclerotic disease in patients with chronic kidney disease.* Nephrology, dialysis, transplantation : official publication of the European Dialysis and Transplant Association - European Renal Association, 2010. **25**(6): p. 1882-8.
11. Go, A.S., et al., *Chronic kidney disease and the risks of death, cardiovascular events, and hospitalization.* The New England journal of medicine, 2004. **351**(13): p. 1296-305.
12. Tonelli, M., et al., *Chronic kidney disease and mortality risk: a systematic review.* Journal of the American Society of Nephrology : JASN, 2006. **17**(7): p. 2034-47.
13. Van Biesen, W., et al., *The glomerular filtration rate in an apparently healthy population and its relation with cardiovascular mortality during 10 years.* European heart journal, 2007. **28**(4): p. 478-83.

14. Liu, J.E., et al., *Association of albuminuria with systolic and diastolic left ventricular dysfunction in type 2 diabetes: the Strong Heart Study.* Journal of the American College of Cardiology, 2003. **41**(11): p. 2022-8.
15. Hemmelgarn, B.R., et al., *Relation between kidney function, proteinuria, and adverse outcomes.* JAMA : the journal of the American Medical Association, 2010. **303**(5): p. 423-9.
16. Muntner, P., et al., *Traditional and nontraditional risk factors predict coronary heart disease in chronic kidney disease: results from the atherosclerosis risk in communities study.* Journal of the American Society of Nephrology : JASN, 2005. **16**(2): p. 529-38.
17. Kestenbaum, B., et al., *Serum phosphate levels and mortality risk among people with chronic kidney disease.* Journal of the American Society of Nephrology : JASN, 2005. **16**(2): p. 520-8.
18. Mourad, J.J., et al., *Creatinine clearance, pulse wave velocity, carotid compliance and essential hypertension.* Kidney international, 2001. **59**(5): p. 1834-41.
19. McMahon, L.P., S.D. Roger, and A. Levin, *Development, prevention, and potential reversal of left ventricular hypertrophy in chronic kidney disease.* Journal of the American Society of Nephrology : JASN, 2004. **15**(6): p. 1640-7.
20. Edwards, N.C., et al., *Aortic distensibility and arterial-ventricular coupling in early chronic kidney disease: a pattern resembling heart failure with preserved ejection fraction.* Heart, 2008. **94**(8): p. 1038-43.
21. Blacher, J., et al., *Impact of aortic stiffness on survival in end-stage renal disease.* Circulation, 1999. **99**(18): p. 2434-9.
22. Ilyas, B., et al., *Renal function is associated with arterial stiffness and predicts outcome in patients with coronary artery disease.* QJM : monthly journal of the Association of Physicians, 2009. **102**(3): p. 183-91.
23. Coca, S.G., et al., *Underrepresentation of renal disease in randomized controlled trials of cardiovascular disease.* JAMA : the journal of the American Medical Association, 2006. **296**(11): p. 1377-84.
24. Mann, J.F., et al., *Renal insufficiency as a predictor of cardiovascular outcomes and the impact of ramipril: the HOPE randomized trial.* Annals of internal medicine, 2001. **134**(8): p. 629-36.
25. Rahman, M., et al., *Renal outcomes in high-risk hypertensive patients treated with an angiotensin-converting enzyme inhibitor or a calcium channel blocker vs a diuretic: a report from the Antihypertensive and Lipid-Lowering Treatment to Prevent Heart Attack Trial (ALLHAT).* Archives of internal medicine, 2005. **165**(8): p. 936-46.
26. Sever, P.S., et al., *Prevention of coronary and stroke events with atorvastatin in hypertensive patients who have average or lower-than-average cholesterol concentrations, in the Anglo-Scandinavian Cardiac Outcomes Trial--Lipid Lowering Arm (ASCOT-LLA): a multicentre randomised controlled trial.* Lancet, 2003. **361**(9364): p. 1149-58.

27. Tonelli, M., et al., *Effect of pravastatin on cardiovascular events in people with chronic kidney disease.* Circulation, 2004. **110**(12): p. 1557-63.
28. *MRC/BHF Heart Protection Study of cholesterol lowering with simvastatin in 20,536 high-risk individuals: a randomised placebo-controlled trial.* Lancet, 2002. **360**(9326): p. 7-22.
29. Jafar, T.H., et al., *Proteinuria as a modifiable risk factor for the progression of non-diabetic renal disease.* Kidney international, 2001. **60**(3): p. 1131-40.
30. Barnett, A.H., et al., *Angiotensin-receptor blockade versus converting-enzyme inhibition in type 2 diabetes and nephropathy.* The New England journal of medicine, 2004. **351**(19): p. 1952-61.
31. Kunz, R., et al., *Meta-analysis: effect of monotherapy and combination therapy with inhibitors of the renin angiotensin system on proteinuria in renal disease.* Annals of internal medicine, 2008. **148**(1): p. 30-48.
32. Di Zhang, A., et al., *Cross-talk between mineralocorticoid and angiotensin II signaling for cardiac remodeling.* Hypertension, 2008. **52**(6): p. 1060-7.
33. Iglarz, M., et al., *Involvement of oxidative stress in the profibrotic action of aldosterone. Interaction wtih the renin-angiotension system.* American journal of hypertension, 2004. **17**(7): p. 597-603.
34. Delcayre, C. and B. Swynghedauw, *Molecular mechanisms of myocardial remodeling. The role of aldosterone.* Journal of molecular and cellular cardiology, 2002. **34**(12): p. 1577-84.
35. Young, M.J., *Mechanisms of mineralocorticoid receptor-mediated cardiac fibrosis and vascular inflammation.* Current opinion in nephrology and hypertension, 2008. **17**(2): p. 174-80.
36. Pitt, B., et al., *The effect of spironolactone on morbidity and mortality in patients with severe heart failure. Randomized Aldactone Evaluation Study Investigators.* The New England journal of medicine, 1999. **341**(10): p. 709-17.
37. Pitt, B., et al., *Eplerenone, a selective aldosterone blocker, in patients with left ventricular dysfunction after myocardial infarction.* The New England journal of medicine, 2003. **348**(14): p. 1309-21.
38. Ezekowitz, J.A. and F.A. McAlister, *Aldosterone blockade and left ventricular dysfunction: a systematic review of randomized clinical trials.* European heart journal, 2009. **30**(4): p. 469-77.
39. Bomback, A.S., et al., *Change in proteinuria after adding aldosterone blockers to ACE inhibitors or angiotensin receptor blockers in CKD: a systematic review.* American journal of kidney diseases : the official journal of the National Kidney Foundation, 2008. **51**(2): p. 199-211.
40. Navaneethan, S.D., et al., *Aldosterone antagonists for preventing the progression of chronic kidney disease: a systematic review and meta-analysis.* Clinical journal of the American Society of Nephrology : CJASN, 2009. **4**(3): p. 542-51.
41. Edwards, N.C., et al., *Effect of spironolactone on left ventricular mass and aortic stiffness in early-stage chronic kidney disease: a randomized controlled trial.* Journal of the American College of Cardiology, 2009. **54**(6): p. 505-12.

42. Heshka, J., et al., *Spironolactone for difficult to control hypertension in chronic kidney disease: an analysis of safety and efficacy.* Journal of the American Society of Hypertension : JASH, 2010. **4**(6): p. 295-301.
43. Svensson, M., et al., *How prevalent is hyperkalemia and renal dysfunction during treatment with spironolactone in patients with congestive heart failure?* Journal of cardiac failure, 2004. **10**(4): p. 297-303.
44. Tamirisa, K.P., K.D. Aaronson, and T.M. Koelling, *Spironolactone-induced renal insufficiency and hyperkalemia in patients with heart failure.* American heart journal, 2004. **148**(6): p. 971-8.
45. Committee, J.F., ed. *British National Formulary*. 62 ed. September 2011, BMJ Group and Pharmaceutical Press: London.
46. Wei, L., et al., *Spironolactone use and renal toxicity: population based longitudinal analysis.* BMJ, 2010. **340**: p. c1768.
47. Compendium, E.M. *Electronic Medicines Compendium on Spironolactone tablets 25mg*. 2nd February 2011; Available from: http://www.medicines.org.uk/EMC/medicine/24182/SPC/Spironolactone+Tablets+25mg/.
48. Antoniou, T., et al., *Trimethoprim-sulfamethoxazole induced hyperkalaemia in elderly patients receiving spironolactone: nested case-control study.* BMJ, 2011. **343**: p. d5228.

18 PROTOCOL AMENDMENTS

Version 1.0	Approved on 22nd March 2012
Version 2.0	Approved on 18th June 2012
Version 3.0	Approved on 26th July 2012

Appendix A

STOP- CKD Hyperkalaemia and Renal Dysfunction Management Protocol

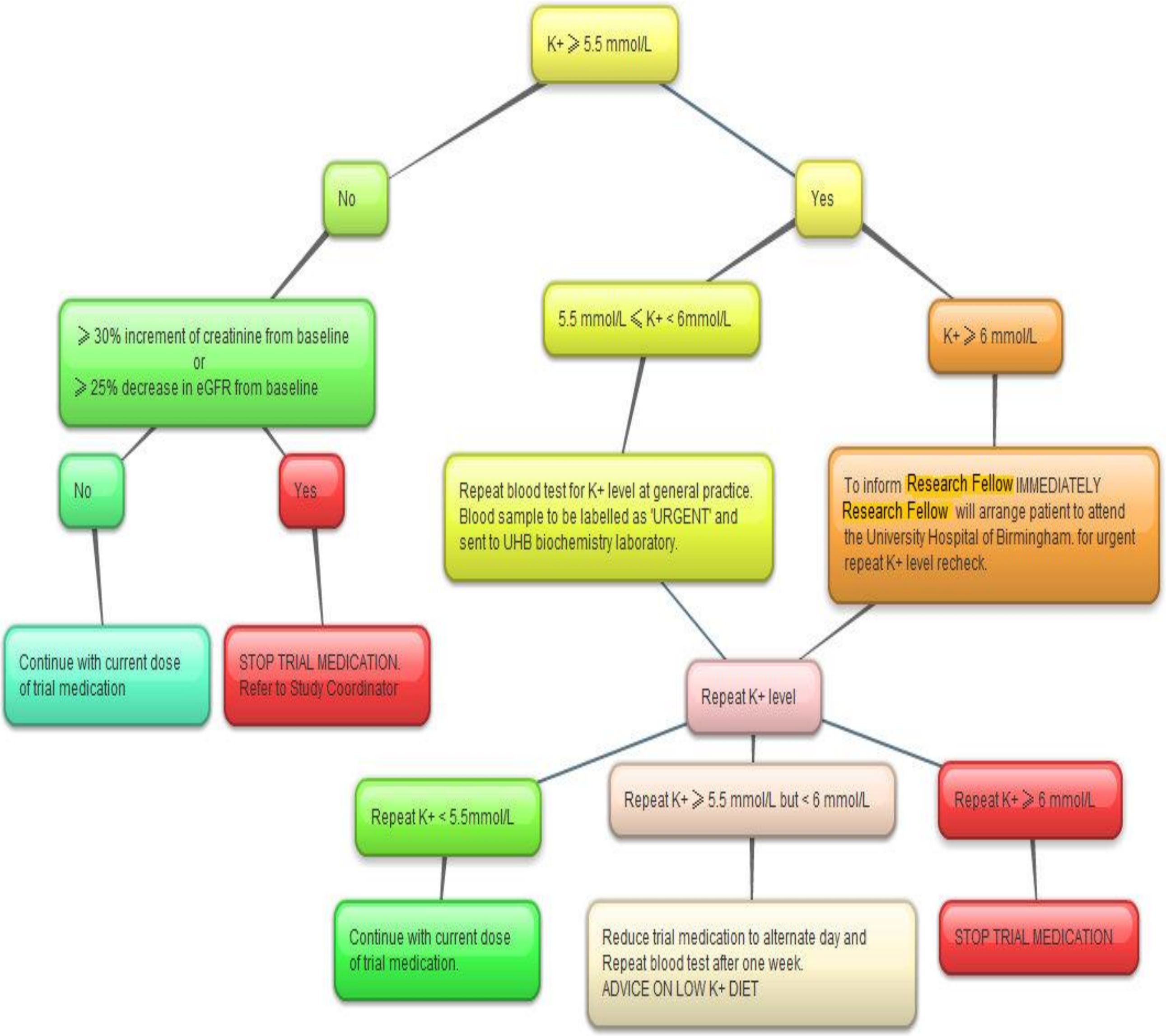

Appendix B: Patient Information Sheet

Appendix C: Consent form

Appendix D

Low Potassium Diet Information Sheet

Potassium is a mineral found in the blood. The level of potassium in the blood should be kept between 3.5-5.5 mmol/L. The trial medication you are receiving may increase the potassium level in your body. High potassium level can be dangerous and affect your heart.

To reduce the level of potassium in your blood, you may need to cut down on certain foods which are high in potassium. This table gives a simple guide on some of the foods to limit and provides tips on alternatives that can be used.

Limit or Avoid	Alternatives you can choose from
**Fruit:**<br>Bananas, strawberries, mango, fresh grapefruit, pineapple, grapes, rhubarb, oranges, kiwi, prunes, ALL dried fruit	*(limit to 2 portions per day)*<br>Apples, pears, Satsuma, tinned fruit drained of juice.
**Vegetables:**<br>Tomatoes, mushrooms, spinach, sweet corn, beetroot, plantains, parsnips, aubergine, broccoli, avocado, baked beans.	*(limit to 2 portions per day)*<br>ALL boiled vegetables, carrots, cabbage, cauliflower, onion, turnip, asparagus, lettuce, cucumber, courgette, celery.
**Potatoes and alternatives: (see below)**<br>Roast, baked or chips (unless the potatoes have been boiled first)	Boiled potatoes, rice, noodles, bread
**Drinks:**<br>Fruit and vegetables juices, smoothies, fruit-juice-based squashes (i.e.: Ribena, Hi-Juice etc).<br>Coffee<br>Beer, cider, lager, sherry, port.	Fizzy drinks without fruit juice and some squashes.<br>Tea<br>Spirits (Gin, Whisky, Vodka, etc).
**Snacks:**<br>**ALL** potato crisps. **ALL** nuts.<br>Chocolate, marzipan, liquorice, toffee.	Snacks made from wheat, corn or rice (i.e.: rice crackers, popcorn, Doritos, Wotsits, Skips). Plain biscuits and cakes, crumpets.<br>Boiled sweets, fruit pastilles, mints, chewing gum.
**Others:**<br>Lo-salt and other salt substitutes	Use other herbs and spices

Milk: limit to ½ pint per day
Potatoes: Potatoes are high in potassium and should be prepared in the following way to reduce their potassium content.

1. Cut in small pieces.
2. Boil in large volume of water.
3. May then be fried, roasted or creamed.

The water used to cook potatoes and vegetables should be discarded.

Appendix E: EQ5D-5L Form

Under each heading, please tick the ONE box that best describes your health TODAY

MOBILITY

I have no problems in walking about ❑
I have slight problems in walking about ❑
I have moderate problems in walking about ❑
I have severe problems in walking about ❑
I am unable to walk about ❑

SELF-CARE

I have no problems washing or dressing myself ❑
I have slight problems washing or dressing myself ❑
I have moderate problems washing or dressing myself ❑
I have severe problems washing or dressing myself ❑
I am unable to wash or dress myself ❑

USUAL ACTIVITIES *(e.g. work, study, housework, family or leisure activities)*

I have no problems doing my usual activities ❑
I have slight problems doing my usual activities ❑
I have moderate problems doing my usual activities ❑
I have severe problems doing my usual activities ❑
I am unable to do my usual activities ❑

PAIN / DISCOMFORT

I have no pain or discomfort ❑
I have slight pain or discomfort ❑
I have moderate pain or discomfort ❑
I have severe pain or discomfort ❑
I have extreme pain or discomfort ❑

ANXIETY / DEPRESSION

I am not anxious or depressed ❑
I am slightly anxious or depressed ❑
I am moderately anxious or depressed ❑
I am severely anxious or depressed ❑
I am extremely anxious or depressed ❑

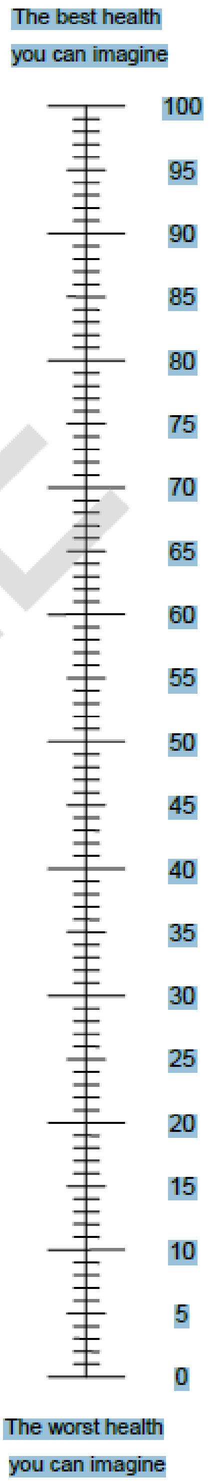

- We would like to know how good or bad your health is TODAY.
- This scale is numbered from 0 to 100.
- 100 means the <u>best</u> health you can imagine.
  0 means the <u>worst</u> health you can imagine.
- Mark an X on the scale to indicate how your health is TODAY.
- Now, please write the number you marked on the scale in the box below.

YOUR HEALTH TODAY =

SAMPLE

Appendix F

Medication Monitoring Questionnaire:

Side Effect	Yes	No
Nausea		
Vomiting		
Abdominal Discomfort		
Diarrhoea		
Black Discoloured Stool		
Tiredness		
Headache		
Confusion		
Drowsiness		
Dizziness/ Imbalance		
Breast swellings		
Breast pain		
Menstrual (period) disturbance		
Change in libido		
Excessive hair growth		
Unwanted hair growth		
Hair loss		
Leg cramps		
Rash		
Joint pain		
Others: (Please comment)		

Have you missed any trial medication since the last visit? Yes / No
If Yes, how many tablets have you missed?

To be completed by healthcare professional

Side Effect	Yes	No
Hyperkalaemia		
Hyponatremia		
Increment of serum creatinine ≥ 30% from baseline or reduction of eGFR ≥ 25% from baseline		
Leucopenia		
Agranulocytosis		
Thrombocytopenia		
Hepatotoxicity		

Appendix G:

Spironolactone Medicine Information

Summary of Product Characteristics last updated on the eMC: 02/02/2011
Contraindications

Spironolactone therapy is contraindicated in the following:

• Anuria (patients are at greater risk of developing hyperkalaemia)

• Active renal insufficiency, rapidly progressing or severe impairment of renal function (spironolactone may aggravate electrolyte imbalance and the risk of developing hyperkalaemia is increased)

• Hyperkalaemia (spironolactone may further increase serum potassium concentrations)

• Addison's disease

• Hypersensitivity to spironolactone or any of the ingredients in the product

• Diabetes mellitus, especially in patients with confirmed or suspected renal insufficiency

• Diabetic nephropathy (increased risk of hyperkalaemia. Spironolactone should be discontinued at least 3 days prior to a glucose tolerance test because of the risk of severe hyperkalaemia).

Special warnings and precautions for use

• Patients with rare hereditary problems of galactose intolerance, the lapp lactase deficiency or glucose – galactose malabsorption should not take this medicine.

• Patients receiving spironolactone should be carefully evaluated for possible disturbances of fluid and electrolyte balance, particularly in the elderly and in those with significant renal and hepatic impairment.

• Hyperkalaemia may occur in patients with impaired renal function or excessive potassium intake and can cause cardiac irregularities which may be fatal. Should hyperkalaemia develop, spironolactone should be discontinued, and if necessary, active measures taken to reduce the serum potassium to normal. Dilutional hyponatraemia may be induced especially when spironolactone is concurrently administered with other diuretics.

• Care should be taken in patients suffering from hyponatraemia.

• Reversible increases in blood urea have been reported with spironolactone therapy, particularly in the presence of impaired renal function.

• Reversible hyperchloraemic metabolic acidosis, usually in association with hyperkalaemia has been reported to occur in some patients with decompensated hepatic cirrhosis, even in the presence of normal renal function.

• Caution is required in severely ill patients and those with relatively small urine volumes who are at greater risk of developing hyperkalaemia.

• Caution is required in patients with a predisposition to metabolic or respiratory acidosis. Acidosis potentiates the hyperkalaemic effects of spironolactone and spironolactone may potentiate acidosis.

• Spironolactone has been shown to produce tumours in rats when administered at high doses over a long period of time. The significance of these findings with respect to clinical use is not certain. However, the long-term use of spironolactone in young patients requires careful consideration of the benefits and the potential hazard involved.

• Caution should be exercised in patients diagnosed with porphyria as spironolactone is considered unsafe in these

patients.

• Care should be taken in patients suffering from menstrual abnormalities or breast enlargement.

Interaction with other medicinal products and other forms of interaction

• ACE inhibitors - since ACE inhibitors decrease aldosterone production they should not routinely be used with spironolactone, particularly in patients with marked renal impairment.

• Angiotensin-II receptor antagonists – concurrent administration of angiotensin-II receptor antagonists, e.g. valsartan, losartan, and spironolactone may result in an increase in serum potassium levels. If concurrent use is necessary, monitor serum potassium levels.

• Antihypertensives - potentiation of the effect of antihypertensive drugs occurs and their dosage may need to be reduced when spironolactone is added to the treatment regime, and then adjusted as necessary.

• Anti-diabetics – administration with chlorpropamide may increase risk of hyponatraemia.

• Aspirin - may reduce the diuretic effect of spironolactone.

• Cardiac glycosides - spironolactone has been reported to increase serum digoxin concentration and to interfere with certain serum digoxin assays. In patients receiving digoxin and spironolactone, the digoxin response should be monitored by means other than serum digoxin concentrations, unless the digoxin assay used has been proven not be affected by spironolactone therapy. If it proves necessary to adjust the dose of digoxin, patients should be carefully monitored for evidence of enhanced or reduced digoxin effect.

• Ciclosporin - co-administration of potassium-sparing diuretics with ciclosporin may result in hyperkalaemia. Avoid concurrent use of spironolactone and ciclosporin. If concurrent therapy is necessary, monitor serum potassium levels for persistent elevations in patients.

• Corticosteroids - co-administration of spironolactone with fludrocortisone may result in a paradoxical dose-related increase in urinary potassium excretion. If concomitant administration is necessary, closely monitor serum potassium levels.

• Coumarins - in patients receiving oral anticoagulant therapy with warfarin, the prothrombin time ratio or INR (international normalised ratio) should be monitored with the addition and withdrawal of treatment with spironolactone, and should be reassessed periodically during concurrent therapy. Adjustments of the warfarin dose may be necessary in order to maintain the desired level of anticoagulation.

• Diuretics - spironolactone should not be administered concurrently with other potassium- sparing diuretics as this may induce hyperkalaemia. Potassium canrenoate, a metabolite of spironolactone, has been shown to cause myeloid leukaemia in rats.

• Lithium - concurrent use of lithium and spironolactone may result in increased lithium concentrations and lithium toxicity (weakness, tremor, excessive thirst, and confusion) due to decreased lithium excretion. If concomitant therapy is necessary monitor serum lithium levels within the first five to seven days of adding or discontinuing spironolactone and periodically thereafter. Lower lithium doses may be required with concomitant spironolactone therapy.

• NSAIDs - may attenuate the natriuretic efficacy of diuretics due to inhibition of intrarenal synthesis of prostaglandins. There may be an increased risk of nephrotoxicity and hyperkalaemia when NSAIDs, notably indometacin are used with spironolactone.

Indometacin and mefenamic acid, inhibit the excretion of canrenone reducing the diuretic effect.

• Potassium salts – potassium supplements are contraindicated except in cases of initial potassium depletion. If potassium supplementation is considered essential, serum electrolytes should be monitored.

• Sympathomimetics - spironolactone reduces vascular responsiveness to noradrenaline (norepinephrine); caution

should be exercised in the management of patients subjected to regional or general anaesthesia.

• Tacrolimus - spironolactone should not be used in patients undergoing therapy with tacrolimus as concomitant use has resulted in mild to severe hyperkalaemia.

• Ulcer healing drugs - as carbenoxolone may cause sodium retention and thus decrease the effectiveness of spironolactone, concurrent use of the two agents should be avoided.

• In fluorimetric assays spironolactone may interfere with the estimation of compounds with similar fluorescence characteristics.

• Liver function tests – spironolactone may enhance the metabolism of antipyrine used in liver function tests.

Pregnancy and lactation

Spironolactone or its metabolites may cross the placental barrier. With spironolactone feminisation has been observed in male rat foetuses. Spironolactone should be used with caution in pregnant women, weighing the potential risk to the mother and foetus against the possible benefits. Canrenone, a metabolite of spironolactone, appears in breast milk, therefore an alternative method of infant feeding should be instituted.

Effects on ability to drive and use machines

Patients should be warned that they may experience dizziness or drowsiness when taking this medicine. They should make sure they are not affected before driving or operating machinery.

Undesirable effects

• *Blood and lymphatic system disorders:* agranulocytosis, eosinophilia and thrombocytopenia have been reported rarely. Spironolactone may cause transient elevations in blood urea nitrogen (BUN) especially in patients with renal impairment. Hyponatraemia has been reported rarely.

• *Hypersensitivity:* these occur rarely and are usually mild but very occasionally may be severe causing swelling, shock and collapse. Shortness of breath, skin rash or itching has been reported rarely.

• *Metabolism and nutrition disorders:* hyperkalemia and hyponatraemia has been reported rarely. Electrolyte disturbances.

• *Nervous system disorders:* ataxia, drowsiness, dizziness, headache and clumsiness have been reported although these are less common.

• *Psychiatric disorders:* lethargy.

• *Cardiac disorders:* severe hyperkalaemia may result in paralysis, flaccid paraplegia and cardiac arrhythmias with subsequent cardiovascular collapse. This can be fatal in patients with impaired renal function.

• *Hepato – biliary disorders:* hepatotoxicity has been reported.

• *Gastrointestinal disorders:* gastritis, gastric bleeding, stomach cramps, diarrhoea, vomiting and ulceration are more frequent effects.

• *Skin and subcutaneous tissue disorders:* urticaria and alopecia has been reported rarely. Skin rashes have also been reported.

• *Musculoskeletal, connective tissue and bone disorders:* osteomalacia.

• *Renal and urinary disorders:* acute renal failure, particularly in those with pre-existing renal impairment.

• *Reproductive system and breast disorders:* gynaecomastia may develop in association with the use of spironolactone. Development appears to be related to both dosage level and duration of therapy and is usually reversible once therapy is discontinued. In rare instances some breast enlargement may persist. Alteration in voice pitch may also occur on rare occasions which may not be reversible. Impotence and decreased sexual ability has been reported. This is usually

reversible on discontinuation of spironolactone. Breast tenderness and increased hair growth in females, irregular menstrual periods and sweating have been reported.

Overdose

Toxic effects of overdosage are drowsiness, mental confusion, nausea, vomiting, dizziness or diarrhoea. Hyponatraemia or hyperkalaemia may be induced but these effects are unlikely to be associated with acute overdosage. Symptoms of hyperkalaemia may manifest as paraesthesia, lassitude and muscular weakness, flaccid paralysis or muscle spasm and may be difficult to distinguish clinically from hypokalaemia.

No specific antidote has been identified. Improvement may be expected on cessation of therapy. Electrocardiographic changes are the earliest specific signs of potassium disturbances. General supportive measures include replacement of fluids and electrolytes may be indicated. For hyperkalaemia, reduce potassium intake, administer potassium-excreting diuretics, intravenous glucose with regular insulin, or oral ion-exchange resins.

PHARMACOLOGICAL PROPERTIES

Pharmacodynamic properties

ATC CODE C03D A01

Spironolactone is a steroid with a structure resembling that of the natural adrenocorticoid hormone, aldosterone. It acts as a competitive inhibitor of aldosterone and acts on the distal portion of the renal tubule thereby increasing sodium and water excretion and reducing potassium excretion. It is classed as a potassium sparing diuretic or aldosterone antagonist.

Pharmacokinetic properties

Absorption – Spironolactone is incompletely but fairly rapidly absorbed from the gastrointestinal tract and the extent of absorption will depend on the particle size and formulation and is improved after food. Bioavailability is estimated from 60 to 90%. Time to peak plasma concentration is approximately one hour.

Distribution – Although the plasma half life of spironolactone itself is short (1.3 hours) the half lives of the active metabolites are longer (ranging from 2.8 to 11.2 hours).

Spironolactone is estimated to be 90% protein bound. Volume of distribution, extent of tissue accumulation and ability to cross the blood brain barrier are not known. Spironolactone or its metabolites may cross the placental barrier and canrenone is secreted breast milk. Spironolactone is known to have a slow onset of action two to three days and a slow diminishment of action.

Metabolism – The main site of biotransformation is the liver where it is metabolised, to 80% sulphur containing metabolities such as 7 alpha- thiomethylspironolactone and canrenone (20%). Many of these metabolities also have a diuretic-activity. Canrenone, which is an active metabolite, has a biphasic plasma half life of about 4 - 17 hours.

Elimination – Spironolactone is excreted in the urine and faeces in the form of metabolites.

The renal action of a single dose of spironolactone reaches its peak after 7 hours, and activity persists for at least 24 hours.

Preclinical safety data

Carcinogenicity spironolactone has been shown to produce tumours in rats when administered at high doses over a long period of time. The significance of these findings with respect to clinical use in not certain. However the long term use of spironolactone in young patients requires careful consideration of the benefits and the potential hazard involved. Spironolactone or its metabolities may cross the placental barrier. With spironolactone, feminisation has been observed in male rat foetuses. The use of spironolactone in pregnant women requires that the anticipated benefit be weighed against the possible hazards to the mother and foetus.

Appendix H: Role and Responsibilities

Brief Study Title: STOP-CKD
Study Start Date: May 2012

Trial Role	Name	Responsibilities	Signature and Date
**Chief Investigator**	Dr Charles Ferro	Lead investigator<br><br>Responsible for the design, conduct, analyses and reporting of STOP-CKD.	
**Co Investigator**	Prof Richard McManus	Expert Primary care input<br><br>Provide advice in the design and conduct of the study.	
	Dr Paramjit Gill	Expert Primary care input<br><br>Provide advice in the design and conduct of the study.	
	Dr John Townend	Specialist cardiology input	
	Dr Sheila Greenfield	Sociology input.<br>Supervision of qualitative study.	
	Dr Poorva Jain	Specialist nephrology input	
**Statistician**	Prof Nick Freemantle	Provide statistical advice and supervise statistical analysis	
**Research Fellow**	Dr Khai Ping Ng	Trial Management<br>Day to day renal advice<br>Training research nurses and trial administrator.	
**Research Nurse**	Gurdip Heer	Day to day running of STOP-CKD study	

**Study Administrator/ coordinator**			

Appendix I: TRIAL PERSONNEL CONTACT SHEET

Brief Study Title: STOP-CKD

Study Start Date: May 2012

Personnel	Name	Address	Telephone	E-mail
**Sponsor**	Brendan Laverty	University of Birmingham<br>Edgbaston<br>Birmingham<br>B15 2TT	0121 414 7618	b.w.laverty@bham.ac.uk
**Chief Investigator**	Dr Charles Ferro	Consultant Nephrologist<br>University Hospital Birmingham<br>Mindelsohn Way<br>Birmingham<br>B15 2WB	0121 3715839	Charles.ferro@uhb.nhs.uk
**Co Investigator**	Prof Richard McManus	Professor of Primary Care Cardiovascular Research<br>Department of Primary Care Health Sciences<br>University of Oxford<br>2nd Floor<br>23-38 Hythe Bridge Street<br>Oxford<br>OX1 2ET.	01865 617834	richard.mcmanus@phc.ox.ac.uk
	Dr Paramjit Gill	Reader in Primary Care Research<br><br>Primary Care Clinical Sciences<br>School of Health and Population Sciences<br>College of Medical and Dental Sciences<br>University of Birmingham	0121 414 3758	p.s.gill@bham.ac.uk
	Dr John Townend	Consultant Cardiologist/ Senior Lecturer<br><br>University Hospital Birmingham<br>Mindelsohn Way<br>Birmingham<br>B15 2WB.	0121 6978476	john.townend@uhb.nhs.uk

Appendix I: TRIAL PERSONNEL CONTACT SHEET

	Dr Sheila Greenfield	Senior Lecturer in Medical Sociology<br><br>Primary Care Clinical Science<br>University of Birmingham<br>Edgbaston<br>Birmingham<br>B15 2TT	0121 4146493	s.m.greenfield@bham.ac.uk
	Dr Poorva Jain	Renal Medicine Research Fellow/Registrar in Nephrology<br><br>Primary Care Clinical Science<br>University of Birmingham<br>Edgbaston<br>Birmingham<br>B15 2TT	0121 4145643	p.jain@bham.ac.uk
**Statistician**	Prof Nick Freemantle	Professor of Clinical Epidemiology and Biostatistics<br><br>Primary care clinical sciences<br>University College London<br>Gower Street<br>London<br>WC1E 6BT	020 77940500 Ext 34756	nicholas.freemantle@ucl.ac.uk
**Research Fellow**	Dr Khai Ping Ng	Renal Medicine Research Fellow/Registrar in Nephrology<br><br>University Hospital Birmingham<br>Mindelsohn Way<br>Birmingham<br>B15 2WB	0800 9230329	k.p.ng@bham.ac.uk
**Research Nurse**	Gurdip Heer	Primary Care Research Nurse<br><br>Primary Care Clinical Science<br>University of Birmingham<br>Edgbaston<br>Birmingham<br>B15 2TT	0 121 4146046	g.k.heer@bham.ac.uk

Appendix I: TRIAL PERSONNEL CONTACT SHEET

**Study Administrator/ coordinator**	To be confirmed			

Appendix J: Pharmacovigilance Flowchart

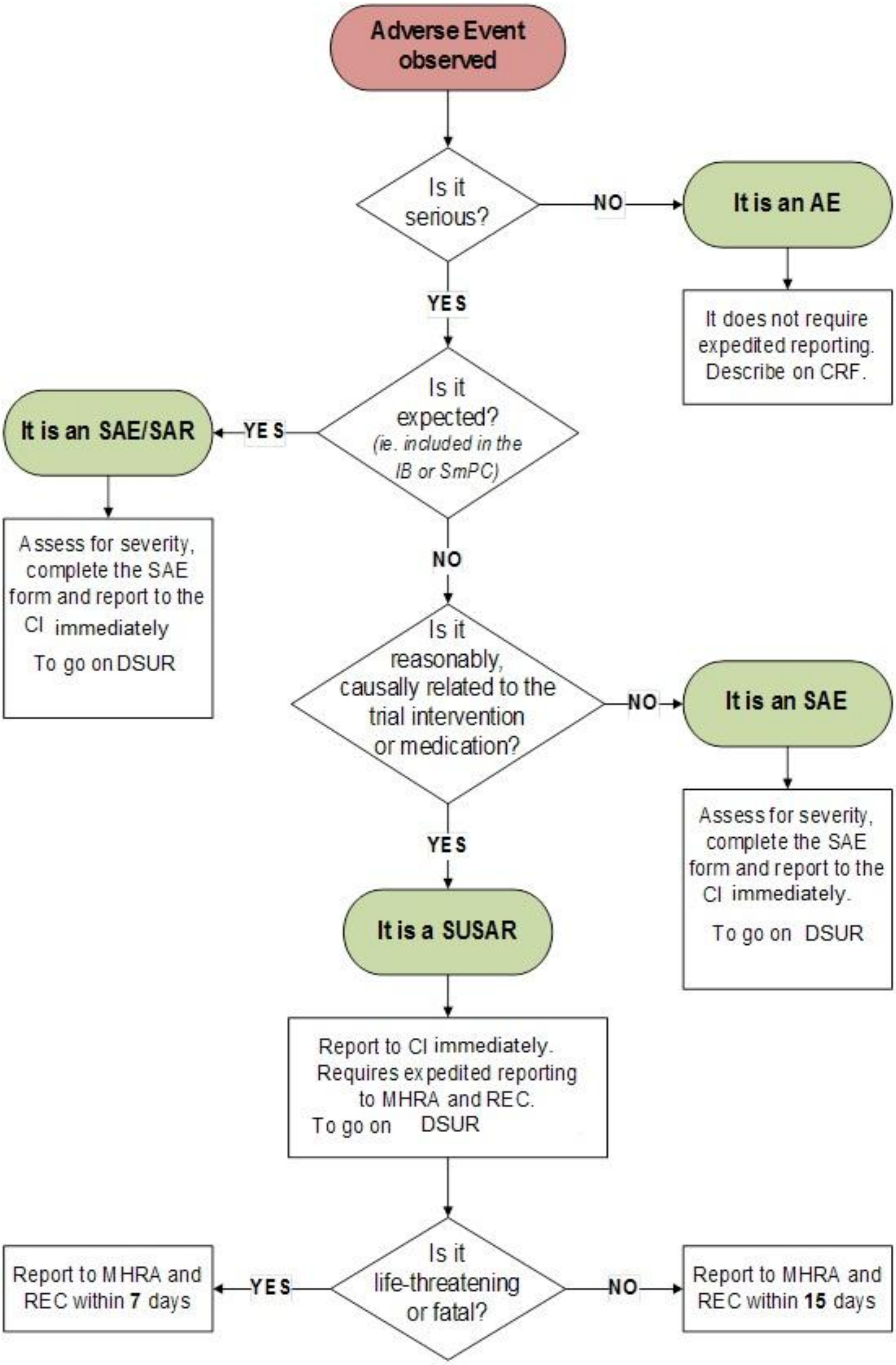

Appendix J: Pharmacovigilance Flowchart
