# Supplementary material for: Results and lessons from the Spironolactone To Prevent Cardiovascular Events in Early Stage Chronic Kidney Disease (STOP-CKD) randomised controlled trial
Source: BMJ Open. 2016 Feb 25;6(2):e010519. doi: 10.1136/bmjopen-2015-010519 (PMC4769397; doi:10.1136/bmjopen-2015-010519)
Supplement: Supplementary data [file bmjopen-2015-010519supp2.pdf]

[GP Name, Address and Contact Number]

[Date as postmark]

Version 2 20/06/2013

Invitation to Participate in Research Study:

<u>STOP-CKD: Spironolactone to Prevent Cardiovascular Events in Early Stage Chronic Kidney Disease: A Pilot Trial</u>

Dear [Title and surname],

We are working with the Primary Care Clinical Research and Trials Unit at the University of Birmingham on a study that aims to look into the possible benefits of the use of a 'water-tablet', called Spironolactone in patients with early stage chronic kidney disease. We are writing to people from the practice to ask for their help and you have been selected as you have had a blood test in the past indicating you may have a lowered kidney function.

We would be very grateful if you would read the attached information sheet about the study and think about whether you would like to take part. Please indicate on the response slip whether or not you are interested in participating in the main study and/or the interview study. A FREEPOST envelope is enclosed for you to return your response directly to the research team at the University.

<u>If you have any questions about the study then you can contact the research team directly on 0800 923 0329</u>.

Thank you for your time.
Yours sincerely,

[Signature]

[Name]
[Practice Lead GP]
